# Dual‐Action Psoriasis Therapy: Antiproliferative and Immunomodulatory Effects via Self‐Locking Microneedles

**DOI:** 10.1002/advs.202409359

**Published:** 2024-10-30

**Authors:** Zi Yi Wang, Ze Qiang Zhao, Yu Jun Sheng, Ke Jun Chen, Bo Zhi Chen, Xin Dong Guo, Yong Cui

**Affiliations:** ^1^ China‐Japan Friendship Hospital (Institute of Clinical Medical Sciences) Chinese Academy of Medical Sciences & Peking Union Medical College Beijing 100029 China; ^2^ Department of Dermatology China‐Japan Friendship Hospital Beijing 100029 China; ^3^ State Key Laboratory of Organic‐Inorganic Composites Beijing University of Chemical Technology Beijing 100029 China; ^4^ Beijing Laboratory of Biomedical Materials College of Materials Science and Engineering Beijing University of Chemical Technology Beijing 100029 China

**Keywords:** antiproliferation, deucravacitinib, immunology, microneedle, psoriasis

## Abstract

Psoriasis is a chronic, immune‐mediated disorder characterized by immune regulation disorders and abnormal keratinocyte proliferation. Deucravacitinib (Deu), a selective oral Tyrosine Kinase 2 (TYK2) inhibitor, shows promise in treating psoriasis but may cause systemic side effects and fail to address persistent localized thickened lesions. Herein, a self‐locking microneedle (MN) patch with a polyvinyl alcohol (PVA) inner ring loaded with Deu is developed, designed to penetrate the transdermal barriers and dissolve rapidly, downregulating the IL‐23/IL‐17 pathway and serve as the first line of defense against the spread of skin‐originated inflammation. Additionally, Calcipotriol (Cal), a vitamin D derivative, is incorporated into a methacrylated hyaluronic acid (HAMA) backing layer and outer ring that mimics occlusive administration, maintaining localized skin surface retention for prolonged anti‐proliferative therapy. The Deu@Cal MN demonstrates satisfactory adhesiveness due to swelling‐mediated mechanical interlocking via the outer ring, ensuring targeted drug release at lesion site. Besides its effectiveness in alleviating both skin inflammation and proliferation, it inhibits the differentiation of Th17 cells in the spleen, suggesting potential to reduce systemic inflammation. These findings offer a new therapeutic approach for treating psoriasis and other autoimmune and inflammatory conditions.

## Graphical abstract

A self‐locking MN patch for psoriasis delivers TYK2 inhibitor to target skin‐originated inflammation and modulate local immune microenvironment, while simultaneously releases Cal for sustained anti‐proliferative effects on persistent hyperproliferative lesions. This dual‐action MN therapy effectively reduces both local and systemic inflammation, presenting a novel therapeutic strategy for autoimmune and inflammatory conditions.

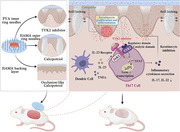

## 1 Introduction

Skin and subcutaneous diseases are prominent contributors to the global burden of nonfatal illnesses. Approximately 0.09%–11.43% of the global population suffers from psoriasis, making it one of the most prevalent chronic skin diseases worldwide.^[^ ^1^ ^]^ Psoriasis is characterized by widespread erythematous, scaly plaques, and itching, and is associated with significant psychosocial comorbidities, including anxiety and depression, suicidal ideation, and substance misuse.^[^ ^2^ ^]^ Pathogenesis involves factors such as heredity, abnormal keratinocyte proliferation and differentiation, and immune dysregulation. Psoriasis is characterized by intricate interactions among infiltrating leukocytes, resident skin cells, and various pro‐inflammatory cytokines produced within the skin, all governed by the cellular immune system. The inflammatory processes that originate in the skin can subsequently lead to systemic inflammation. Thus, effective management of skin inflammation is essential for the treatment of psoriasis and for mitigating its systemic consequences.^[^ ^3^, ^4^ ^]^ Current oral treatments for moderate to severe psoriasis, such as methotrexate, acitretin, and cyclosporine, target widespread intracellular pathways, potentially leading to broad‐ranging effects and various side effects (e.g., bone marrow suppression, liver fibrosis, teratogenicity).^[^ ^5^ ^]^

Deucravacitinib (Deu), a first‐in‐class oral Tyrosine kinase 2 (TYK2) inhibitor, has demonstrated favorable therapeutic outcomes in real‐world clinical practice for the treatment of psoriasis compared to many oral therapies such as methotrexate and apremilast.^[^ ^6^, ^7^ ^]^ Deu stabilizes the TYK2 pseudokinase (JH2) domain through allosteric inhibition, disrupting the interaction between TYK2's regulatory and catalytic domains. This blockade inhibits downstream signal transduction and signal transducers and activators of transcription (STAT)‐dependent gene transcription.^[^ ^8^ ^]^ The Janus kinase (JAK)‐STAT pathway is engaged at various points within the interleukin‐23 (IL‐23)/T helper 17 (Th17) signaling pathway, which is considered central to the pathogenesis of psoriasis.^[^ ^9^ ^]^ Deu may provide an improved therapeutic index and reduce the toxicities associated with pan‐JAK inhibitors, as it demonstrates ≈100‐ to 200‐fold greater selectivity for JAK1/JAK3 and over 3000‐fold greater selectivity for JAK2.^[^ ^10^, ^11^ ^]^

Despite its effectiveness in treating psoriasis, Deu can cause adverse effects such as upper respiratory infections and herpes zoster, and is contraindicated in patients with severe hepatic impairment or latent tuberculosis infection.^[^ ^7^, ^10^, ^12^, ^13^ ^]^ Therefore, developing transdermal formulations of TYK2 inhibitors is essential for psoriasis treatment with reduced systemic exposure, yet such formulations for Deu are currently unavailable.

Although oral TYK2 inhibitor can improve overall clinical manifestations through systemic immune regulation, some patients still experience persistence of localized thickened lesions.^[^ ^6^, ^7^ ^]^ Calcipotriol (Cal), a vitamin D derivative, induces a dose‐dependent decrease in keratinocyte proliferation and promotes terminal differentiation.^[^ ^14^ ^]^ Cal can be locally used to address the limitations of systemic medications in managing localized, stubborn hyperproliferative lesions. Occlusion therapy with Cal effectively reduced the scaliness and thickness of psoriatic lesions by enhancing therapeutic response and drug penetration, facilitated by increased temperature and humidity that improve stratum corneum permeability.^[^ ^15^ ^]^ However, the poor adhesiveness of occlusive materials limits their convenience of use.^[^ ^15^, ^16^ ^]^

**Figure 1:**
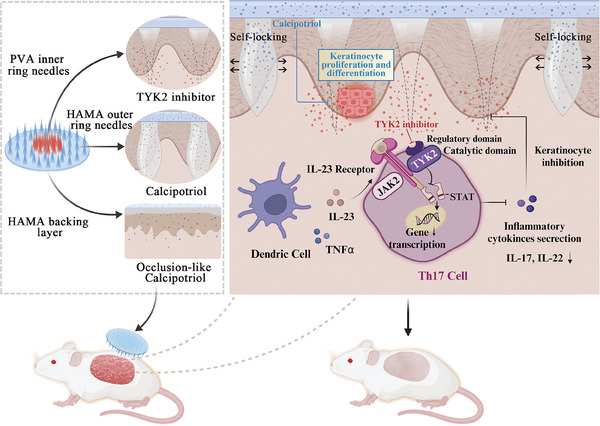
Schematic illustration of design and mechanism of self‐locking Deu@Cal MN‐mediated antiproliferative and immunomodulatory effects for psoriasis therapy.

Based on these considerations, we developed a dual‐release microneedle patch. The polyvinyl alcohol (PVA) needles fully penetrate the transdermal barriers to rapidly release the TYK2 inhibitor in a single‐dose manner, effectively mimicking “localized oral administration.” This approach modulates the local immune microenvironment of the lesions, serving as a primary defense against the propagation of skin‐originated systemic inflammation. Concurrently, Cal is stationed in the outer ring of the microneedle patch and backing layer for sustained anti‐proliferative therapy on the surface of psoriatic skin (**Figure** **1**). The occlusion‐like Cal administration could in turn promote Deu penetration through scaly and thickened lesions. To enhance packaging adhesion and facilitate clinical application, we developed swelling‐mediated mechanical interlocking via the outer ring material to ensure targeted drug release at the lesion site. In vivo experiments conducted in our study demonstrated that Deu@Cal MN effectively downregulated psoriasis‐associated IL‐23/IL‐17 pathways and alleviated epidermal hyperplasia, thereby achieving a combined synergistic effect of immunomodulation and antiproliferation on the local lesions. Additionally, Deu@Cal MN inhibits the differentiation of Th17 cells in the spleen, suggesting its potential to mitigate systemic inflammation. Our findings present a novel therapeutic strategy for treating psoriasis and other autoimmune and inflammatory conditions.

## 2 Results and Discussion

### 2.1 Synthesis and Characteristics of Deu@Cal MNs

**Figure 2:**
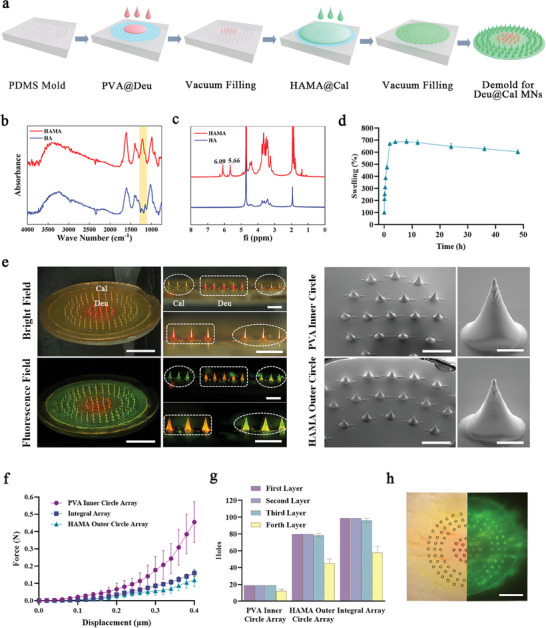
Preparation and characteristics of Deu@Cal MNs. a) Schematic diagram of the microneedle preparation process. b) FTIR, c) 1H NMR, and d) dissolution property evaluation of HAMA. e) Deu@Cal MNs in microscope bright field, fluorescence field and SEM topography (Scale bar: 5 mm, 1 mm, 100 µm). Evaluation of mechanical properties of MNs, f) mechanical displacement curves, g) sealing film piercing performance, and h) pig skin piercing test (Scale bar: 3 mm).

Using a template vacuum filling method, solutions containing different drug matrix materials were filled into distinct regions of the mold to fabricate microneedle patches with dual drug release capabilities (**Figure** **2**a). These patches improved3 adhesion through swelling‐mediated mechanical interlocking via the outer ring material. Methacrylated hyaluronic acid (HAMA) was selected as the outer ring material due to its superior biocompatibility, swelling behavior, and photopolymerization properties, which are ideal for Deu@Cal MN fabrication. To confirm the synthesis of HAMA, its molecular structure was analyzed using Fourier‐transform infrared (FTIR) spectroscopy and hydrogen nuclear magnetic resonance (^1^HNMR) spectroscopy. The FTIR spectrum of hyaluronic acid (HA) exhibited an ─OH stretching vibration peak at 3220 cm⁻¹ and a bending vibration peak at 1400 cm⁻¹. Following the reaction, the product exhibited strong peaks at 1610 and 1220 cm⁻¹, corresponding to C═O stretching and C─O─C stretching vibrations, respectively, indicating ester bond formation and successful grafting of methacrylic anhydride (MA) onto the HA molecular chain (Figure 2b). The ^1^HNMR spectrum revealed two distinct proton peaks at chemical shifts of 5.66 and 6.09, characteristic of the double bonds in the grafted methacrylate (Figure 2c). Upon photopolymerization and soaking in phosphate‐buffered saline (PBS), HAMA rapidly absorbed water and swelled, achieving a swelling ratio exceeding 650% within 2 h (Figure 2d). After freeze‐drying, the material exhibited a microporous structure with pore sizes in the hundreds of micrometers (Figure S1, Supporting Information), validating its superior swelling performance to ensure rapid mechanical interlocking and improved adhesion and encapsulation of Cal upon insertion.

Microscopic examination revealed that the Deu@Cal MN array maintained its structural integrity. The array consisted of conical needles measuring 600 µm in height and 300 µm in base diameter, arranged in a circular pattern. Rhodamine B (red) representing Deu was primarily distributed within the inner ring PVA needles, while calcein (green) representing Cal was found in the HAMA backing layer and needles (outer ring) (Figure 2e). Scanning Electron Microscopy (SEM) analysis demonstrated that the needle surfaces were smooth, with no evident drug precipitation, and had sharp tips, ensuring effective skin penetration. The mechanical displacement curve and in vitro insertion performance tests confirmed the Deu@Cal MN's ability to penetrate the stratum corneum, thereby enhancing drug absorption and delivery efficiency. The material composition significantly influenced mechanical performance; although HAMA exhibited lower mechanical strength compared to PVA, it still generated sufficient force (0.06 N per needle at 0.32 mm displacement) to meet the minimum skin insertion requirement (Figure 2f). Parafilm M film insertion tests demonstrated that all microneedle types could penetrate three layers of film (each 125 µm thick) (Figure 2g). Fluorescent model drugs were utilized to evaluate the penetration efficiency of the microneedles into porcine skin. As expected, the Deu@Cal MN arrays successfully penetrated the skin, forming distinct fluorescent insertion points (Figure 2h). Deu@Cal MNs demonstrated exceptional biocompatibility, as indicated by the negligible hemolysis observed during co‐incubation with blood. The hemolysis rate was consistently below 2%, with red blood cells remaining intact and unruptured (Figure S2, Supporting Information). Furthermore, when fibroblasts were co‐incubated with varying concentrations of Deu@Cal MNs for 24 h, the cells exhibited high viability. AM‐PI staining confirmed the absence of significant cell death (Figure S3, Supporting Information), underscoring the superior biocompatibility of Deu@Cal MNs.

### 2.2 Characterization of Mechanical Embedding and Encapsulation Properties of Deu@Cal MNs

**Figure 3:**
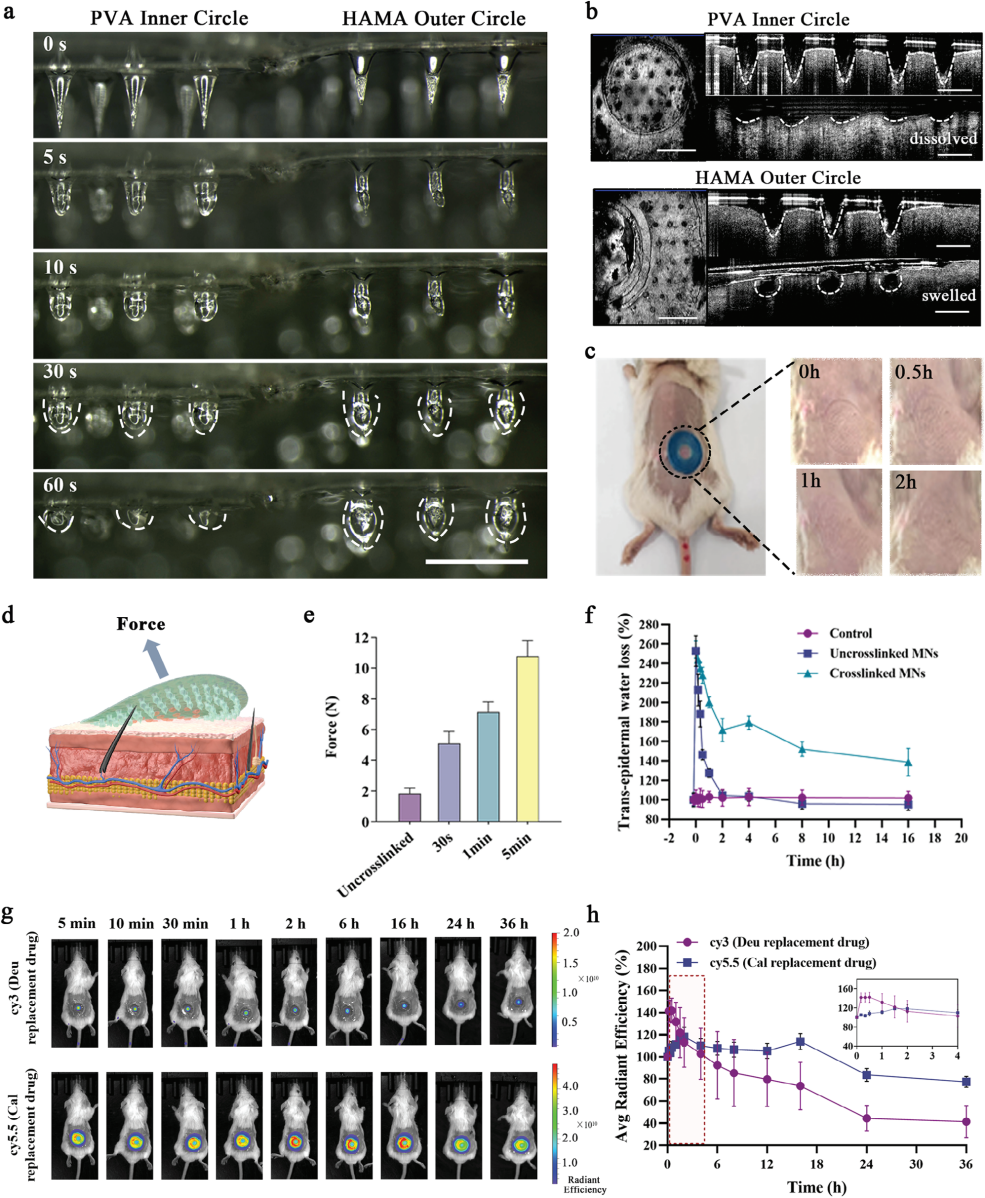
Characterization of mechanical embedding and encapsulation properties of Deu@Cal MNs. a) In vitro microneedle morphological and structural changes in high humidity environment (Scale bar: 1 mm). b) OCT imaging of the structural changes of microneedle puncture into the subcutaneous skin of mice (Scale bar: 2mm, 500 µm). c) Changes in the recovery of Deu@Cal MNs insertion sites on the skin surface of mice. d) Schematic illustration of mechanical embedding and e) evaluation of adhesion force of Deu@Cal MNs. f) Change in skin surface moisture before and after MNs treatment. g) Representative in vivo fluorescence images of mice at different time points after administration of the respective simulated fluorescent drugs. h) Relationship between mean square displacement and time for Cy3 (substitute for Deu) and Cy5.5 (substitute for Cal) (*n *= 3).

The dual‐release behavior of the microneedle patches is characterized by the rapid dissolution of the PVA needles in the inner ring, which are loaded with Deu. Upon subcutaneous insertion, PVA needles quickly dissolved, releasing Deu to inhibit the regulatory domains of TYK2. Concurrently, the HAMA needles in the outer ring swell upon insertion, forming a mechanical interlocking structure that enhances patch adhesion and enables the sustained release of Cal. To visualize the structural characteristics of the Deu@Cal MNs, they were placed in a 95% humidity environment, where the inner ring PVA dissolved rapidly within 60 s, while the outer ring HAMA gradually swelled as it absorbed water (**Figure** **3**a). In vivo behavior of the microneedles in mice confirmed these observations. Optical coherence tomography (OCT) imaging can serve as a non‐invasive alternative to histopathology, providing insights into the longitudinal structure of the skin. The OCT images showed that the needle tips of both the PVA inner circle and HAMA outer circle of the Deu@Cal MNs penetrate beneath the basal layer. However, post‐insertion images revealed that the PVA component of the inner ring dissolved nearly completely within 5 min, resulting in closure of the channels. Simultaneously, the HAMA component swelled, forming a spherical interlocking structure under the skin, thereby enhancing mechanical adhesion (Figure 3b). The micro‐holes created by Deu@Cal MNs were largely healed within 2 h after removal without inducing local allergic reactions such as erythema (Figure 3c). Tensile testing to measure the minimum separation force between the microneedle patch and the skin showed that the cross‐linked HAMA increased the separation force from 1.84 ± 0.35 to 5.11 ± 0.78 N following subcutaneous insertion, with further increases over time (Figure 3d,e). The improved adhesion was attributed to the subcutaneous mechanical interlocking structure formed by the swollen HAMA, which enhanced adhesion as its volume expanded over time.

The transepidermal water loss (TEWL) exhibited a very slow decline in cross‐linked MNs (Figure 3f), indicating their capacity to maintain microchannels in the stratum corneum during the drug delivery phase, thereby facilitating prolonged delivery of Cal and achieving extended drug release. Furthermore, due to the packaging effect of MNs on the skin surface, the increased humidity within the MN system further enhanced effective drug penetration and therapeutic outcomes.^[^ ^15^ ^]^ To further validate the drug release behavior mediated by the Deu@Cal MNs and to infer drug absorption kinetics and duration, thereby guiding subsequent dosing protocols, Cyanin 3 (Cy3) was employed as a surrogate for Deu and Cy5.5 for Cal. The changes in corresponding fluorescence signals were monitored using a small animal in vivo imaging system. The fluorescence signal from the inner ring representing Deu exhibited a gradual decrease as the PVA dissolved and the drug was metabolized and absorbed, with the signal diminishing to ≈40% of its initial intensity within 24 h. In contrast, the encapsulated Cal released through the swelling channels of HAMA and backing layer showed a sustained and slow absorption profile, maintaining a relatively stable fluorescence signal over time. This indicated a prolonged release of Cal on the skin surface, supporting long‐term therapeutic effects on the lesions, similar to localized occlusive therapy. (Figure 3g,h; Figure S4, Supporting Information).

### 2.3 Therapeutic Effect of Deu@Cal MNs in Skin Lesion of Psoriatic Mouse Model

**Figure 4:**
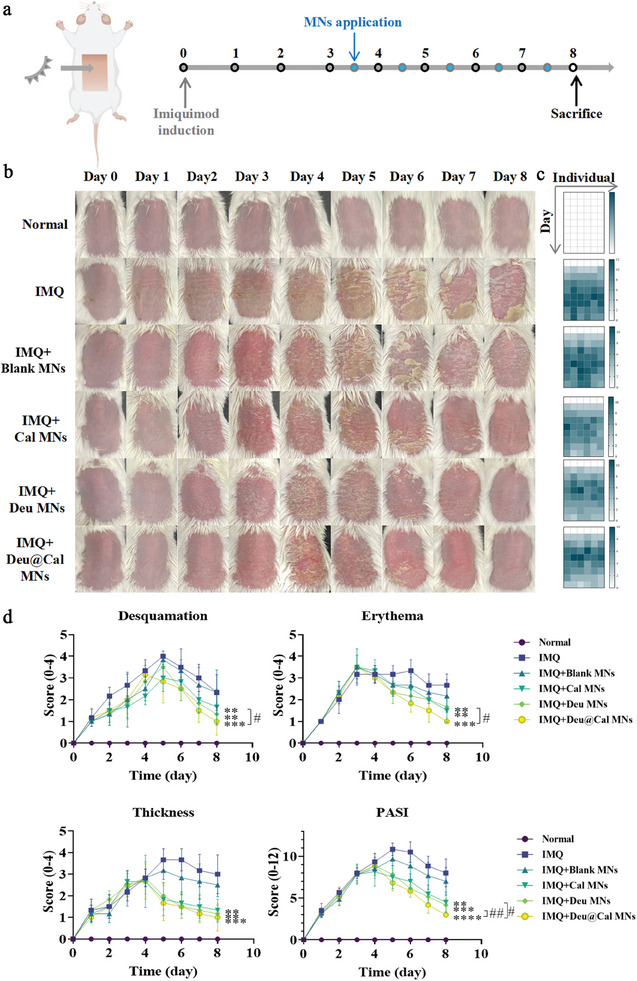
Therapeutic effect of Deu@Cal MNs in mice psoriatic lesions. a) Schematic illustration of Deu@Cal MNs for the treatment of IMQ‐induced psoriatic mice. b) Representative images of mice dorsal skins in different groups from days 0 to 8 (*n* = 6 for each group). c) Heatmap of PASI score (total) of each individual. d) The PASI scores including desquamation, erythema, thickness and total score of different treatment were recorded from days 0 to 8, each score ranged from 0 to 4, and the total score from 0 to 12. (*n *= 6, ** *p *< 0.01, *** *p *< 0.001, and **** *p *< 0.0001 versus IMQ group, ^#^ *p *< 0.05 and ^##^ *p *< 0.01. Data are presented as mean ± SD).

The in vivo therapeutic efficacy of MNs was assessed using an imiquimod (IMQ)‐induced psoriatic mouse model.^[^ ^17^ ^]^ IMQ was applied to the mouse skin for 8 consecutive days from day 0, and MN patches were applied every day from Day 3 (**Figure** **4**a). The IMQ‐induced psoriasis‐like mice were randomly divided into four treatment groups (*n *= 6): Blank MNs, Cal MNs, Deu MNs, and Deu combined with Cal MNs (Deu@Cal MNs). These groups were compared with a normal group and a model group (untreated IMQ group).

The model group exhibited typical physiological characteristics of psoriasis, including white scales, erythema, and thickened skin. After 5 days of treatment, both the Deu MNs and Cal MNs groups showed significant therapeutic effects on psoriasis, with the Deu@Cal MNs group demonstrating the most pronounced relief of symptoms (Figure 4b). The Psoriasis Area and Severity Index (PASI), which assesses erythema, scaling, and skin thickening as indicators of skin inflammation, was used to evaluate psoriasis severity throughout the experiment period.^[^ ^18^ ^]^ Mice treated with Deu@Cal MNs showed significant alleviation in erythema by day 4 and day 5, improved skin thickness by day 5, and reduced desquamation by day 6, while the untreated psoriasis group began returning to normal around day 8. Blank MNs exhibited minimal therapeutic effects compared to the untreated group, suggesting that MNs alone have limited ability to reverse the disease. PASI scores and heatmaps were consistent with the symptoms observed in images, indicating that Deu@Cal MNs proved most effective in alleviating typical psoriatic symptoms, particularly in erythema and desquamation scores. (Figure 4c,d).

Furthermore, the biosafety of Deu@Cal MN in vivo was evaluated through monitoring body weight and examining organ histology. Throughout the experimental period, mice in all treatment groups showed no significant weight loss. (Figure S5, Supporting Information). The histological analysis of heart, liver, spleen, lung, and kidney did not show obvious tissue injuries (Figure S6, Supporting Information). The excellent biosafety profile of Deu@Cal MNs highlights their promising clinical potential.

### 2.4 In Vivo Antiproliferation Effect of Deu@Cal MNs

**Figure 5:**
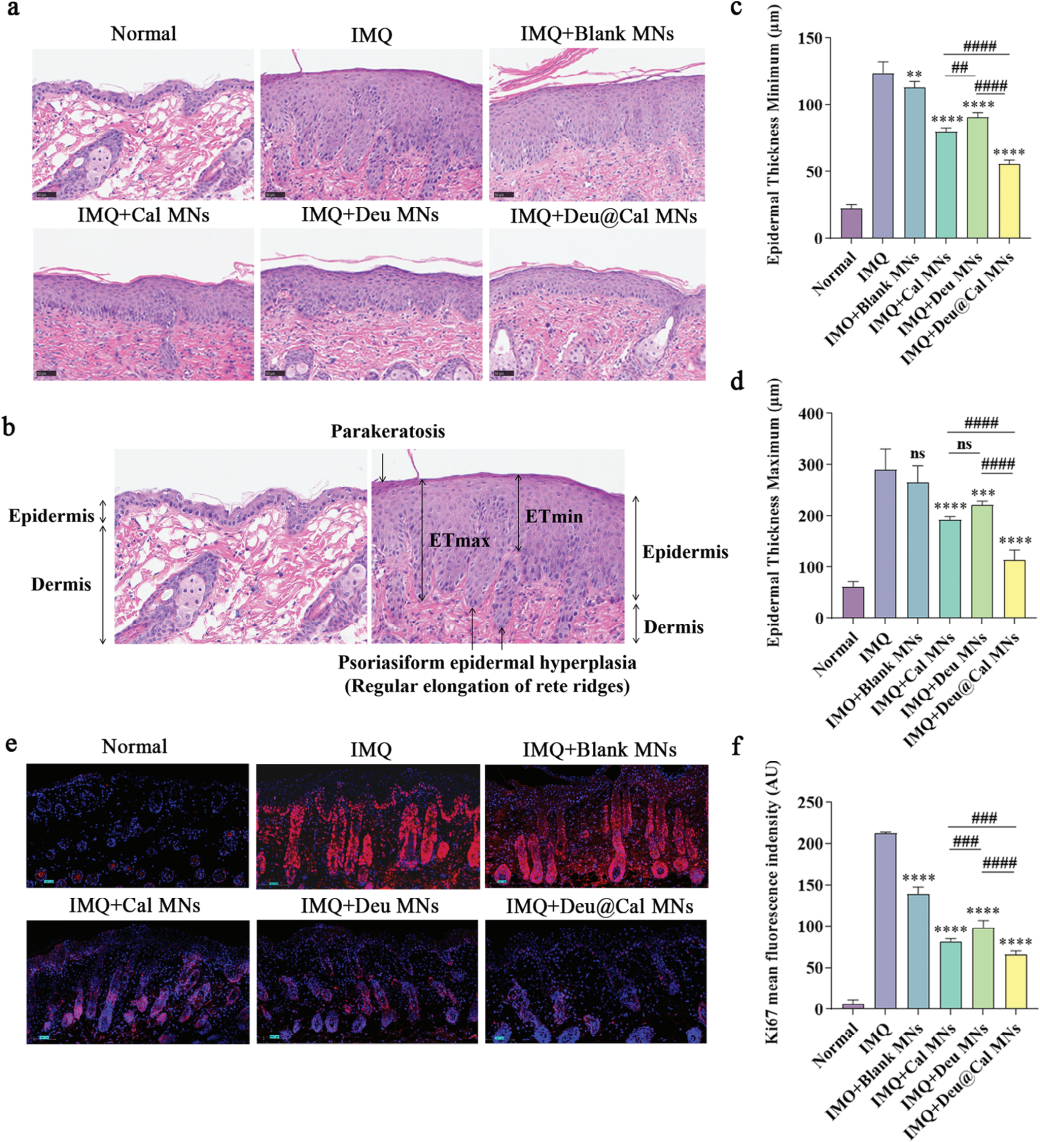
In vivo antiproliferation effect of Deu@Cal MNs. a) Hematoxylin and eosin staining of the mice dorsal skin after different treatments on day 8. b) Histopathologic illustration of ETmin and ETmax in psoriatic lesions. c) ETmin with different treatments on day 8. d) ETmax with different treatments on day 8^th^. e) Immunofluorescence staining of the Ki67 (Scale bar: 100 µm). f) Mean fluorescence intensity of Ki67 of different groups. (* *p *< 0.05, ** *p *< 0.01, and *** *p *< 0.001 **** *p *< 0.0001 versus IMQ group, ^##^ *p *< 0.01, ^###^ *p *< 0.001, and ^####^ *p *< 0.0001. Data are presented as mean ± SD. ns, not significant).

Psoriasis‐like mice and those treated with blank MNs exhibited typical histopathological features of psoriasis, including hyperkeratosis, parakeratosis, psoriasiform epidermal hyperplasia, and inflammatory cell infiltration.^[^ ^19^ ^]^ Treatment with Cal MNs markedly reduced epidermal thickness, however, obvious acanthosis and slightly elongated rete ridges were still observed in Deu MNs group. Notably, Deu@Cal MNs restored epidermal structure to nearly normal levels, with regular epidermal layers. (**Figure** **5**a).

Due to the undulated structure of the dermoepidermal junction (DEJ) in psoriasis, we used the parameters Epidermal Thickness Minimum (ETmin) and Epidermal Thickness Maximum (ETmax) to measure thickness from the top to the bottom of the dermal papillae, respectively (Figure 5b).^[^ ^20^ ^]^ ETmin and ETmax in Deu@Cal MN‐treated group were significantly decreased compared to untreated group (55.8 µm vs 123.8 µm, and 113.8 µm vs 290.6 µm respectively, *p *< 0.0001), indicating substantial alleviation of acanthosis and psoriasiform hyperplasia in psoriatic lesions. Remarkably, Cal MNs exhibited a more pronounced effect on acanthosis compared to Deu MNs, with ETmin values of 79.9 ± 2.6 µm versus 90.6 ± 3.4 µm (*p *< 0.01) (Figure 5c,d). Although both Cal MNs and Deu MNs alleviated epidermal thickening, the mitigation effects of Deu@Cal MNs on hyperplasia were significantly superior, consistent with measurements of dorsal skin thickness in mice (Figure S7, Supporting Information).

Ki67, a nuclear proliferation marker, is indicative of keratinocyte proliferation and reflects the severity of psoriasis.^[^ ^21^ ^]^ Immunofluorescence staining demonstrated that the Cal MN group inhibited Ki67 expression more effectively compared to the Deu MN group (*p *< 0.001), and the Deu@Cal MN group exhibited the lowest expression of Ki67 (Figure 5e,f). These findings were consistent with histopathological observations and in vivo measurements of epidermal thickness.

These results indicated that Cal was more effective than Deu in alleviating epidermal hyperplasia, and this phenomenon agreed well with the prior and our study suggesting Cal decreased proliferation of keratinocytes in vitro (Figure S8, Supporting Information). Occlusion‐like application of Cal in vivo could amplify this effect, demonstrating significant inhibition of hyperplasia when it was combined with Deu.

### 2.5 In Vivo Immunomodulatory Effect in Psoriatic Skin Lesions

The inflammatory environment in psoriatic lesions is characterized by immune cell infiltration and the secretion of various cytokines. Notably, the tumor necrosis factor‐alpha TNF‐α and IL‐23/IL‐17 axis are considered crucial mediators for regulating the functions of dendritic cells and Th17 cells, thereby promoting the development of psoriasis.^[^ ^22^ ^]^ IL‐6 is elevated in the serum and skin lesions of psoriatic patients and may serve as an indicator of inflammatory activity in psoriasis.^[^ ^23^ ^]^

**Figure 6:**
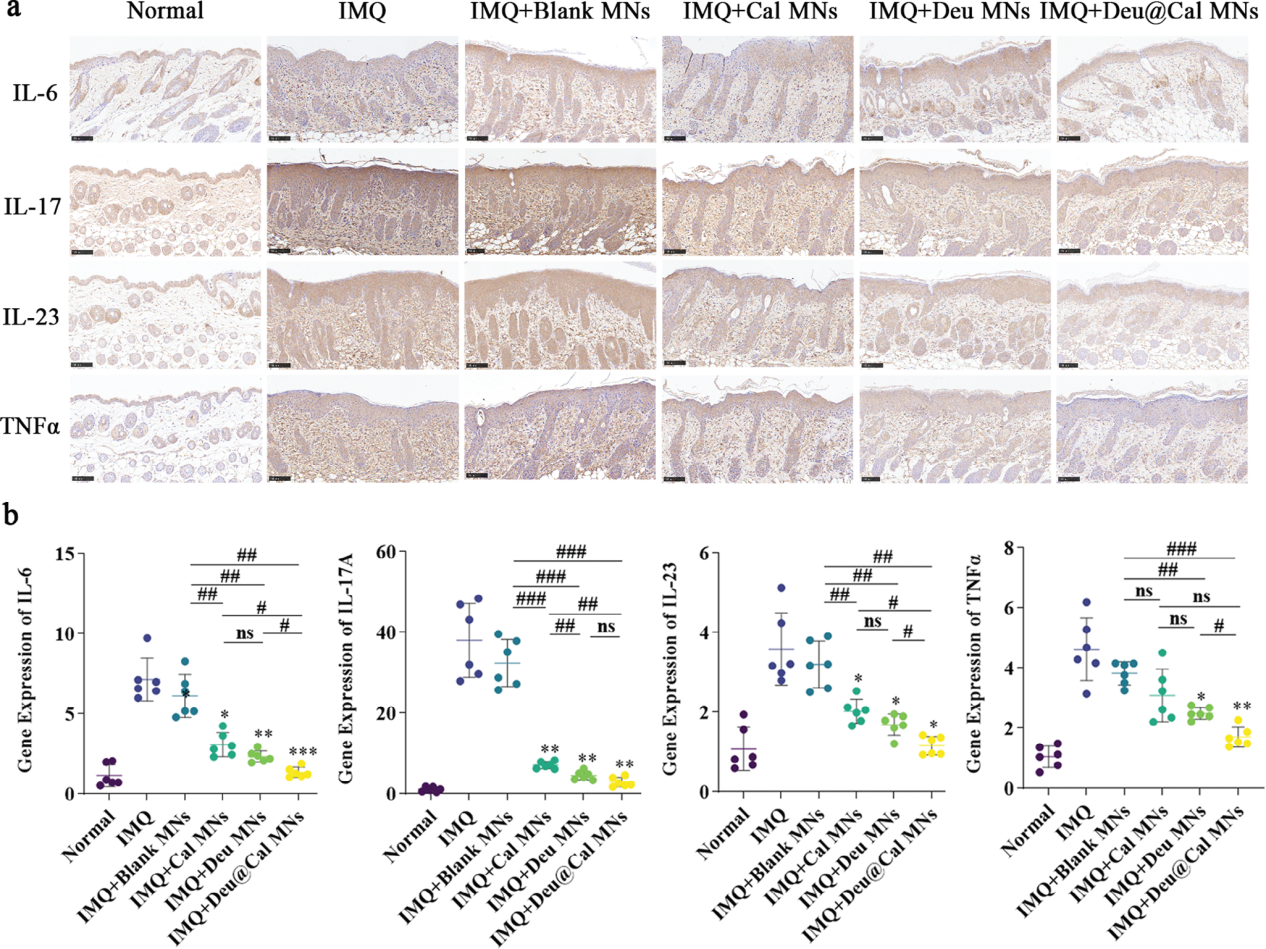
In vivo immunomodulatory effect in psoriatic skin lesions. a) Immunohistochemical staining of IL‐6, IL‐17, IL‐23, and TNF‐α (Scale bar: 100 µm). b) mRNA expression of IL‐6, IL‐17, IL‐23, and TNF‐α in mice dorsal skin measured by qRT‐PCR. (* *p *< 0.05, ** *p *< 0.01, and *** *p *< 0.001 **** *p *< 0.0001 versus IMQ group, ^##^ *p *< 0.01, ^###^ *p *< 0.001, and ^####^ *p *< 0.0001. Data are presented as mean ± SD. ns, not significant).

The immunohistochemistry (IHC) staining results for TNF‐α, IL‐23, IL‐17, and IL‐6 in the skin tissue are shown in **Figure** **6**a. These cytokines exhibited significantly elevated expressions during psoriasis progression and noticeable declines following Deu@Cal MN treatment. Additionally, the mRNA expression levels of these cytokines were measured using quantitative reverse transcription polymerase chain reaction (qRT‐PCR) (Figure 6b). As expected, consistent with the IHC results, the upregulated IL‐17 expression induced by IMQ was significantly more decreased in the Deu MN group compared to the Cal MN group (*p *< 0.01), with the combined Deu@Cal MN therapy showing the most pronounced reduction. The Cal MN group showed no significant difference in TNF‐α levels compared to psoriatic mice, whereas Deu@Cal MN therapy markedly decreased TNF‐α expression (*p *< 0.01). Deu@Cal MN therapy decreased the levels of IL‐23 and IL‐6, the efficacy was significantly higher than that of the Cal MNs (*p *< 0.05). Taken together, Deu@Cal MNs exhibited a satisfactory immunomodulatory effect, with Deu playing a leading role in downregulating the IL‐23/IL‐17 axis, thereby regulating immune responses and inflammation in local lesions.

### 2.6 Evaluation of Spleen Inflammation and Th17 Cell Differentiation

**Figure 7:**
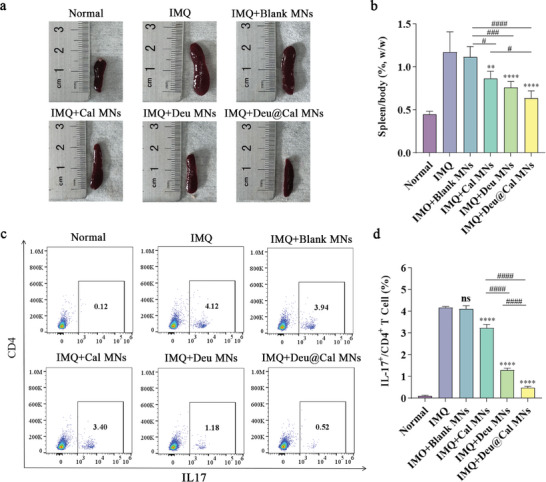
Analysis of splenomegaly and Th17 cells in mice spleen. a) Representative images of mice spleen after different treatments on day 8. b) Spleen/body weight of mice in different groups. c) Representative flow cytometry charts of CD4+IL‐17A+ cells in mice spleen. d) The ratio of CD4+IL‐17A+ cells in spleen analyzed by flow cytometry. (* *p *< 0.05, ** *p *< 0.01, and *** *p *< 0.001 **** *p *< 0.0001 versus IMQ group, ^##^ *p *< 0.01, ^###^ *p *< 0.001 and ^####^ *p *< 0.0001. Data are presented as mean ± SD. ns, not significant).

Given the key role of the spleen in the immune system, it may contribute to psoriasis‐like inflammation by modulating immune responses.^[^ ^24^ ^]^ Previous studies have reported that topical application of IMQ could cause significant splenomegaly, typically regarded as an indication of elevated systemic inflammation.^[^ ^25^ ^]^ We observed that the markedly increased spleen volumes in IMQ‐induced mice were dramatically reduced following Deu@Cal MNs treatment (**Figure** **7**a; Figure S9, Supporting Information), demonstrating the systemic immune adjustment effect of Deu@Cal MNs. Moreover, Deu@Cal MNs significantly decreased the spleen index, whereas the Cal MNs alone had a limited impact on relieving splenomegaly (0.87 vs 0.64, *p *< 0.05) (Figure 7b).

Th17 cells play an essentially immunoregulative role in the pathogenesis of psoriasis by secreting various cytokines, including IL‐17A, IL‐17F, IL‐22, and TNF‐α.^[^ ^26^ ^]^ The Th17 subset, identified as a lineage of CD4+ T cells, primarily produces IL‐17A. In our study, we analyzed the proportion of IL‐17A+ CD4+ T cells in the spleen by flow cytometry and found that the proportion was significantly lower in the Deu@Cal MN group compared to other groups (Figure 7c). Deu@Cal MN treatment achieved substantial inhibition of Th17 cells compared to the IMQ group (0.48% vs 4.17%, *p *< 0.0001). Specifically, the proportion of Th17 cells in the spleen was much lower in the Deu MN group than in the Cal MN group (1.29% vs 2.24%, *p *< 0.0001) (Figure 7d), indicating that Deu exerts a potent immunoregulatory effect at both local and systemic levels. These results suggested that Deu@Cal MN can inhibit spleen inflammation, thereby attenuating systemic psoriasis‐like inflammation.

### 2.7 Therapeutic Mechanisms Based on Transcriptomics

To further elucidate the therapeutic mechanisms underlying the immunoregulatory effects of Deu@Cal MNs in the treatment of psoriasis, the bulk RNA sequencing was conducted to uncover biological functions and molecular mechanisms within psoriatic lesions.

**Figure 8:**
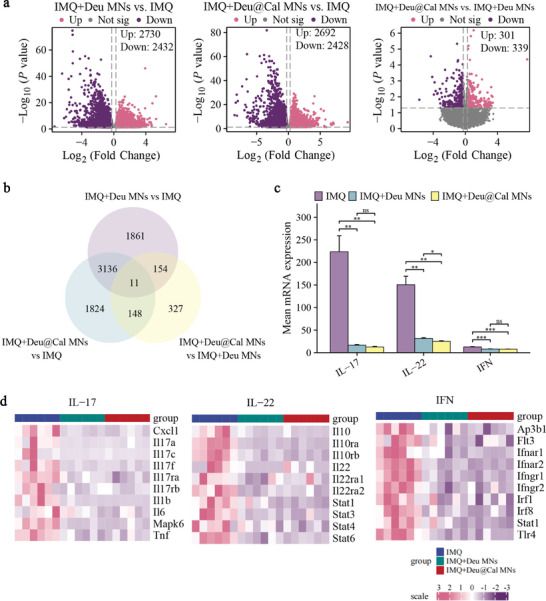
Transcriptome profiling to elucidate genes significantly affected in IMQ, Deu MN, and Deu@Cal MN treatment group by RNA‐Seq. a) Volcano plot showed differentially expressed genes (DEGs) of the IMQ, Deu MN, and Deu@Cal MN treatment group. b) Venn diagram showed the co‐expression of DEGs by comparative analysis of Deu MN versus IMQ, Deu@Cal MN versus IMQ, Deu@Cal MN versus Deu MN. c) Mean mRNA expression associated with type I IFN, IL‐22, and IL‐17 signaling pathways of each group. d) Clustered heat map showed genes that belong to the type I IFN, IL‐22, and IL‐17 signaling pathways that were differentially expressed between Deu@Cal MN, Deu MN, and IMQ group. (* *p *< 0.05, ** *p *< 0.01, and *** *p *< 0.001. Data are presented as mean ± SD. ns, not significant).

A total of 5120 differentially expressed genes (DEGs) were identified in skin treated with Deu@Cal MN compared to the untreated group, with 2692 up‐regulated and 2428 down‐regulated genes (**Figure** **8**a) using 1.2 as the fold change threshold. Venn diagram showed the shared 3147 DEGs between Deu MN versus IMQ and Deu@Cal MN versus IMQ group (Figure 8b). Furthermore, the significantly downregulated genes were highly enriched in type I IFN, IL‐22, and IL‐17 signaling pathways after Deu@Cal MN treatment, which were distinct inflammatory and keratinocyte‐response pathways involved in psoriasis. The mean mRNA expression levels of type I IFN‐associated genes (such as *Ifnr1, Ifnr2, Ifnr1*, and *Ifnr2*, etc.), IL17‐associated genes (*Il17a, Il17f*, and *Il17ra*, etc.) and IL 22 associated genes (*IL22, Il22ra1, Stat1*, and *Stat3*, etc.) were significantly decreased in Deu@Cal MN group compared with IMQ group (*p *< 0.01), and the level of IL‐22 in Deu@Cal MN group was significantly lower than Deu MN treatment alone (*p *< 0.05) (Figure 8c,d).

**Figure 9:**
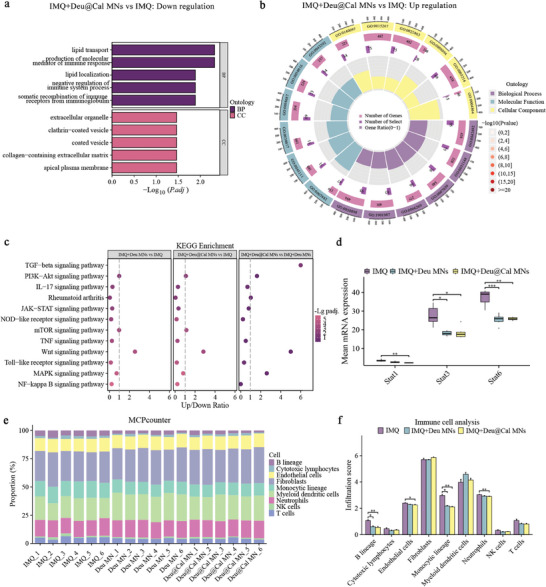
Transcriptome profiling to elucidate pathologic pathways and immune infiltrates in IMQ, Deu MN, and Deu@Cal MN treatment group. a) Gene ontology (GO) enrichment analysis of the down‐regulated DEGs. b) GO enrichment analysis of the up‐regulated DEGs. c) KEGG enrichment analysis of up‐regulated and down‐regulated genes in each comparison. The color from red to purple represents the significant size of the enrichment. d) Mean mRNA expression of Stat1, Stat3 Stat 6 of each group. e) MCP‐counter was used to show the immune composition of each sample. f) The quantification analysis of immune infiltrates. (* *p *< 0.05, ** *p *< 0.01, and *** *p *< 0.001. Data are presented as mean ± SD. ns, not significant).

Gene Ontology (GO) functional annotation and enrichment analyses were conducted to elucidate the biological processes associated with DEGs. Deu@Cal MN treatment had significant effects on down‐regulating production of molecular mediators of immune response, negative regulation of immune system process, and somatic recombination of immune receptors from immunoglobulin in biological processes (BP); and extracellular organelle, coated vessels, and apical plasma membrane in cell components (CC) (**Figure** **9**a). While regulation of cell cycle phase transition, adenosine triphosphate (ATP)‐dependent activity acting on DNA and protein phosphatase inhibitor activity were up‐regulated after Deu@Cal MN therapy (Figure 9b; and Table S1, Supporting Information). Furthermore, Kyoto Encyclopedia of Genes and Genomes (KEGG) pathway enrichment analysis revealed that following Deu MN and Deu@Cal MN treatment, psoriasis‐associated pathways such as the IL‐17, JAK‐STAT, and TNF signaling pathways were significantly down‐regulated due to a higher number of downregulated DEGs compared to upregulated DEGs in each pathway. (Figure 9c). Notably, STAT1, STAT3, and STAT6 expression were substantially inhibited after Deu@Cal MN therapy (Figure 9d), which served as downstream targets of TYK2, previously shown to provide protection against multiple autoimmune and chronic inflammatory disorders.^[^ ^27^ ^]^

Additionally, we calculated immune infiltration scores using microenvironment cell populations counter (MCP‐counter) (Figure 9e), and the quantification analysis showed that neutrophils and monocytic lineage were significantly decreased when treated with Deu@Cal MNs compared with the untreated group (*p *< 0.01) (Figure 9f). Neutrophils, a regulator between the innate and adaptive immune systems, appear early in new psoriatic lesions and contribute to sustained inflammation in psoriasis.^[^ ^28^ ^]^ Moreover, neutrophils are associated with the IL‐17‐producing Th17 subset of CD4 T cells.^[^ ^29^ ^]^ Therefore, Deu@Cal MN therapy effectively downregulated the expression of key genes and pathogenic pathways (e.g., JAK‐STAT signaling pathway) involved in psoriasis, and also reduced associated immune cell infiltration.

## 3 Conclusion

Psoriasis is characterized by epidermal hyperproliferation, abnormal differentiation of epidermal keratinocytes, and inflammation with immunologic alterations in the skin. Thus, treatment targeting the immune microenvironment in local lesions can prevent the spread of skin lesions and systemic complications. In this study, we introduced a self‐locking Deu@Cal MN with satisfactory adhesiveness to ensure targeted drug release at the lesion site. Deu released rapidly to regulate local immune‐mediated cytokine‐initiated inflammation and minimize systemic exposure. Cal showed a sustained release and slow absorption profile, indicatig that Cal provided prolonged anti‐proliferative effect on the the surface of persistent hyperproliferative lesions. Our study demonstrated that Deu@Cal MNs exhibited excellent therapeutic efficacy with a combined synergistic effect of immunomodulation and antiproliferation when treating psoriasis.

IL‐23‐driven Th17 pathways played a crucial role in chronic inflammation in psoriasis and in numerous other inflammatory conditions. Deu significantly downregulated IL‐17 expression in lesions, and inhibited the differentiation of Th17 cells in spleen, indicating the immunoregulation effect on both local and systemic levels. Additionally, in vitro and in vivo studies demonstrated that Cal significantly reduced keratinocyte proliferation, inhibited Ki67 expression, and substantially alleviated acanthosis and psoriasiform hyperplasia in psoriatic lesions. The combination of Cal with Deu results in a more pronounced reduction in lesion thickness, as Deu can indirectly inhibit keratinocyte proliferation by blocking inflammatory cascade responses within the lesions. Notably, the occlusion‐like Cal administration improved humidity and permeability of the stratum corneum, which reduced surface scaling and, in turn, improved Deu penetration and amplifying both therapeutic effects.

Our study demonstrated that Deu@Cal MN treatment effectively targets both adaptive immunity (mediated by IL‐23) and innate immunity (mediated by type I IFN). The downregulated type I IFN‐associated gene expression suggested that Deu@Cal MN therapy may prevent autoimmune cell activation, and treat autoimmune diseases with a predominant role for type I IFN, such as cutaneous lupus.^[^ ^30^ ^]^ The reduced neutrophil infiltration in the lesion suggested that Deu@Cal MNs could inhibit the formation of new lesions, such as those seen in the Koebner phenomenon.^[^ ^31^ ^]^ Moreover, Deu@Cal MN therapy could also regulate the TYK2‐STAT cascade, indicating its protection from multiple autoimmune and inflammatory disorders. In conclusion, Deu@Cal MN hold significant clinical potential for treating autoimmune diseases with skin involvement.

## 4 Experimental Section

### Materials

Polydimethylsiloxane (PDMS, Sylgard 184) was obtained from Dow Corning (Midland, USA). MA was purchased from Bidepharm (Shanghai China). D2O, HA (400 kDa), and PVA (9‐10 kDa) were purchased from Sigma‐Aldrich (MO, USA). Cy3, Cy5.5‐NHS was purchased from Duofluor Inc. (Wuhan China). PBS, Dulbecco's Modified Eagle Medium (DMEM), penicillin‐streptomycin antibiotics, and 0.25% trypsin‐EDTA solution were purchased from Gibco (New York USA). Certified fetal bovine serum (FBS) was purchased from VivaCell (Shanghai China). Cell counting kits (CCK‐8) were purchased from Beijing Solarbio Science & Technology Co., Ltd (Beijing China). Calcein‐AM/PI Double Stain Kit were purchased from Beyotime (Shanghai China). Photoinitiator 2959, Ninhydrin were purchased from Aladdin (Shanghai China). Antibodies involved in immunohistochemistry and immunofluorescence were purchased from Wuhan Servicebio Co., Ltd (Wuhan China).

### Preparation and Characteristics of HAMA

To achieve a composite microneedle structure, HAMA was selected as the base material, following previously reported methods. Specifically, a precise amount of HA was dissolved in PBS and stirred at 600 rpm at 45 °C until fully dissolved. The HA solution was then cooled in an ice‐water bath at 4 °C for 30 min. Subsequently, 2 mL MA was added dropwise at 300 rpm under ice bath conditions, and NaOH was used to adjust the pH to 8.0. The reaction was allowed to proceed for 24 h, yielding the initial product of HAMA. The next day, the reaction mixture was centrifuged to remove any precipitates. The supernatant was transferred to a 12 kDA dialysis bag and dialyzed at room temperature for one week, with the deionized water changed twice daily. After dialysis, the purified product was filtered through a 0.44 µm aqueous microfiltration membrane and lyophilized to obtain HAMA.

To verify the modification, 10.0 mg of HA and HAMA were dissolved in 1.0 mL ultrapure water, vacuum‐dried to form transparent films, and analyzed using a Thermo Fisher FTIR to measure transmittance in the range of 500–4000 cm^−1^. The molecular structure of the products and the degree of substitution of HAMA were determined by dissolving 5.0 mg of HA and HAMA in 500 µL D2O and analyzing with an AV‐600 1H‐NMR.

Additionally, to study the swelling properties of HAMA, a 4% (w/v) HAMA solution containing 0.5% (w/v) Irgacure 2959 was prepared. This solution was poured into a mold (8 mm × 8 mm × 1 mm) to create HAMA patches. After 30 min, the patches were cross‐linked under UV light for 10 s. The patches were then fully dried and weighed, followed by immersion in PBS solution at 37 °C. At various time points, the patches were removed, blotted to remove surface moisture, weighed, and returned to the PBS solution. After 48 h, the patches were freeze‐dried for one day and weighed again. Freeze‐dried samples were also sectioned and the pore morphology observed via SEM. The swelling ratio was calculated using the following equation:

(1) $$\mathrm{Swelling}\;\mathrm{Degrees}\;(\%)=\;\frac{m_{s}-m_{d}}{m_{s}}\times 100$$

where m_s_ was the mass of swollen HAMA patches and m_d_ was the mass of freeze‐dried HAMA patches.

### Preparation and Characteristics of Deu@Cal MNs

To achieve the swelling‐induced mechanical interlocking of microneedles, a custom PDMS mold with an inner and outer ring structure was designed. The inner ring consisted of 19 needles arranged in 1, 6, and 12 needle arrays, while the outer ring comprised 81 needles in 18, 27, and 36 needle arrays. A custom mask was applied to the PDMS mold, and a 25% PVA solution containing Deu was added to the inner ring. The setup was vacuumed for 10 min to remove excess solution, followed by vacuum drying for 30 min. Subsequently, a 4% HAMA solution containing Cal was added to the outer ring, vacuumed for 30 min, and UV cross‐linked for stabilization, followed by an additional 60 min of vacuuming. The mold was dried overnight and demolded using a custom‐made baseplate, then stored in a vacuum desiccator for further characterization and use. Rhodamine B and calcein were used as model drugs to observe drug distribution within the microneedles using brightfield and fluorescence microscopy, with SEM used to examine the integrity and tip morphology of the microneedle arrays.

To ensure the microneedle arrays possessed sufficient mechanical strength for skin insertion, an advanced digital dynamometer (ESM301, Mark‐10, Force Gauge Model, USA) was employed to determine their mechanical and insertion properties. The microneedles were affixed to a test platform, and the force gauge probe descended at a rate of 10 mm min^−1^ to record the force‐displacement curve. Additionally, the microneedles were inserted into Parafilm M film or fresh excised porcine skin with a fixed force of 10N using the force measurement device to observe the insertion performance.

### Characterization of Mechanical Eembedding and Encapsulation Properties of Deu@Cal MNs

The prepared microneedles were placed in an environment with 95% humidity, and changes in the structure of the inner and outer ring needles were observed and recorded at different time points using a handheld microscope. Additionally, the microneedles were inserted into the dorsal skin of mice, and their subcutaneous morphological changes were observed using OCT. After removing the microneedles, the recovery of the needle holes on the mouse skin surface was documented.

To assess the impact of swelling‐mediated mechanical interlocking on adhesion, cross‐linked and non‐cross‐linked microneedles were inserted into porcine skin, and the force required for separation was measured. The water content at the corresponding skin sites was determined using a skin moisture analyzer to compare the drug encapsulation effects of different microneedle patches.

For in vivo drug release analysis, Cy3 was used as a substitute for Deu, and Cy5.5 was used as a substitute for Cal, loading them into the inner and outer rings of the microneedles, respectively. The in vitro transdermal delivery efficiency was evaluated using Franz diffusion cells, with mouse abdominal skin serving as the model. After inserting the Deu@Cal MNs, receptor fluid samples were collected at various time points and analyzed for drug concentration using a fluorescence spectrophotometer, allowing to characterize the drug release profile. In vivo release behavior was assessed using a small animal in vivo imaging system.

### Animals and Psoriasis‐Like Inflammation Model

The female BALB/c mice (6–8 weeks old, 18–22 g) were obtained from SPF (Beijing) Biotechnology Co., Ltd. All the mice were anesthetized with Isoflurane (RWD, China), and a 2.5 × 3 cm area was selected on the back of the mouse and shaved carefully. Each mouse was applied with 62.5 mg IMQ ointment on the prepared back skin once daily in the morning, and MNs treatment at night, except for the control group. Skin thickness was measured once a day, and all mice were killed on the 8th day for follow‐up detection. All animal experiments were approved by the Animal Ethics Committee of China‐Japan Friendship Hospital (CRDWLL 230 155) and were carried out following the National Guidelines for the Care and Use of Laboratory Animals.

### Evaluation of the Severity of Skin Inflammation and Spleen Index

PASI score involved skin erythema, scales, and thickness. Erythema, scales, and thickness were scored independently from 0 to 4, where 0 represents “none,” 1 represents “slight,” 2 represents “moderate,” 3 represents “marked,” and 4 represents “severe.” The total severity of skin inflammation and lesion score was calculated as the sum of the three indexes (0–12). All mice in each group were scored daily for 8 consecutive days from day 0. The body mass and spleen mass of all mice were measured on the seven days. This was used in the calculation of the spleen index using the formula:

(2) spleenindex=spleenmass(mg)/bodymass(g)

### Histological, Immunofluorescence Staining, and Immunohistochemistry Analysis

Dorsal skin samples of all mice were collected and fixed with 4% paraformaldehyde for 24 h, embedded in paraffin, and then sliced into 4 µm thick sections. For histological analysis, the aforementioned thick sections were stained with H&E, and Image‐pro Plus 6.0 was used for the calculation of the epithelial thickness of the skin. The expression of Ki67, IL‐6, IL‐17, IL‐23, and TNF‐α was evaluated by immunofluorescence staining and immunohistochemical staining. The sections were visualized by panoramic scanner (WISLEAP‐10, Jiangsu, China).

### Real‐Time Quantitative PCR

Total mRNA was extracted from biopsies of the dorsal skin isolated after sacrificing the mice using RNAiso Plus (TaKaRa, Japan) according to the manufacturer's instruction, and reverse transcription of mRNA was performed using 4×Hifair III SuperMix plus (Yeasen, Shanghai, China). Through the application of SYBR Green reagent (Yeasen, Shanghai, China), IL‐6, IL‐23, IL‐17A, TNF, and GAPDH mRNA levels were detected by the Gentier 32R Real‐Time PCR System. Sequences for the PCR primers were seen in Table S2 (Supporting information). Gene expression levels in all samples were normalized using the 2^−ΔΔCt^ method with GAPDH as internal controls for comparison.

### Flow Cytometric Assays

The spleen was grinded and filtered using a 40 µm cell strainer to obtain single cell suspension, then was incubated for 5 h in a 37 °C, 5% CO2 incubator. One microliter of Brefeldin A Solution (1000×) (BioLegend, 420 601) was added to each 1 mL of cell suspension to stimulate cells. APC/Cyanine7 anti‐mouse CD45 Antibody (BioLegend,157 203), Pacific Blue anti‐mouse CD3 Antibody (BioLegend, 100 213), and APC anti‐mouse CD4 Antibody (BioLegend, 116 013) were added to lymphocytes for cell staining. For IL‐17A intracellular staining, cells were fixed and permeabilized by Intracellular Fixation/Permeabilization Buffer Kit (Elabscience Biotechnology Co.,Ltd) and FITC anti‐mouse IL‐17A (BioLegend, 506 907) was added. Flow cytometric analysis was performed on the BD LSRFortessa SORP. The data were analyzed by Flowjo software.

### Transcriptome Profiling

For further investigation of therapeutic mechanism, RNA sequencing (RNA‐seq) was performed on lesion skin tissues from IMQ, Deu MNs, and Deu@Cal MNs treatment groups by Novogene Co., Ltd. Data analysis was conducted using DESeq to identify DEGs with a *p*‐value < 0.05 and Fold‐change ≥ 1.2 considered significant. Further analysis included GO enrichment, KEGG pathway enrichment, and MCP‐counter.

### Statistical Analysis

In this study, data were analyzed using GraphPad Prism 8.0 software and presented as means ± standard deviation (M ± SD). One‐way analysis of variance (ANOVA) was then used to analyze the levels of variance within the groups at a significance threshold of *p *< 0.05.

## Conflict of Interest

The authors declare no conflict of interest.

## Author Contributions

Z.Y.W. and Z.Q.Z. contributed equally to this work as co‐first authors. Z.Y.W. and Z.Q.Z. conceived the idea, performed the experiment and drafted the paper. K.J.C. assisted with experiment conduction. Y.J.S. and B.Z.C. designed the overall experimental planning. Z.Y.W. and Z.Q.Z. contributed to the data interpretation. Y.C. and X.D.G. directed the research and provided financial support.

## Supporting information



Supporting Information

## Data Availability

The data that support the findings of this study are available from the corresponding author upon reasonable request.
