# Correction to “Dual‐Action Psoriasis Therapy: Antiproliferative and Immunomodulatory Effects via Self‐Locking Microneedles”

**DOI:** 10.1002/advs.202515046

**Published:** 2025-09-17

*Adv Sci*. 2024;11(48):e2409359.

1) In **Figure 3**g, the images of “Cy5.5 (Cal replacement drug)” at 30min and 1h were placed in the wrong sequence. In addition, the 6h image was incorrectly replaced by the 4h image. We tracked down the original data and have replaced it with the correct image as follows:

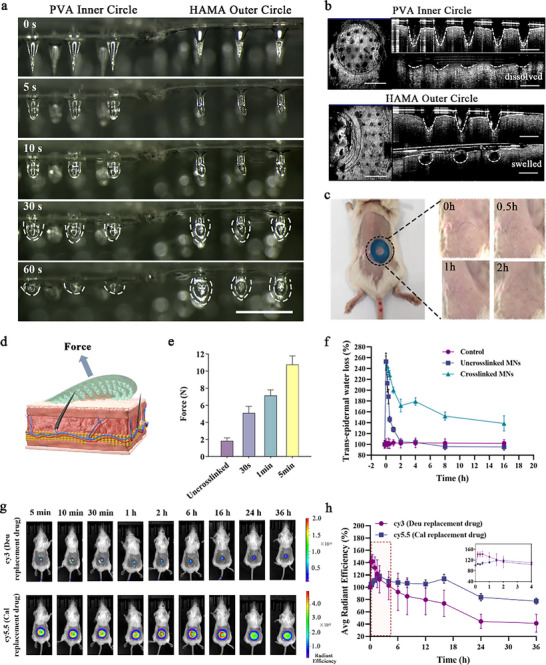


Figure 3. Characterization of mechanical embedding and encapsulation properties of Deu@Cal MNs. a) In vitro microneedle morphological and structural changes in a high‐humidity environment (Scale bar: 1 mm). b) OCT imaging of the structural changes of microneedle puncture into the subcutaneous skin of mice (Scale bar: 2mm, 500 µm). c) Changes in the recovery of Deu@Cal MNs insertion sites on the skin surface of mice. d) Schematic illustration of mechanical embedding and e) evaluation of adhesion force of Deu@Cal MNs. f) Change in skin surface moisture before and after MNs treatment. g) Representative in vivo fluorescence images of mice at different time points after administration of the respective simulated fluorescent drugs. h) Relationship between mean square displacement and time for Cy3 (substitute for Deu) and Cy5.5 (substitute for Cal) (*n* = 3).

2) In **Figure S4** (Supporting Information), the images of “Cy5.5” at 4 and 6h were placed in the wrong sequence. We tracked down the original data and have replaced the correct image as follows:

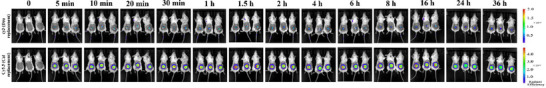


Figure S4. In vivo fluorescence images of mice at different time points after administration of the respective simulated fluorescent drugs. (Cy3 as Deu replacement drug and Cy5.5 as Cal replacement drug, *n* = 3).

3) **Figure S8** (Supporting Information), the images in the second row, corresponding to Cal 1 × 10^−^⁹ mol L^−1^, were inadvertently replaced with images from the Control group. We tracked down the original data and have replaced it with the correct image as follows:

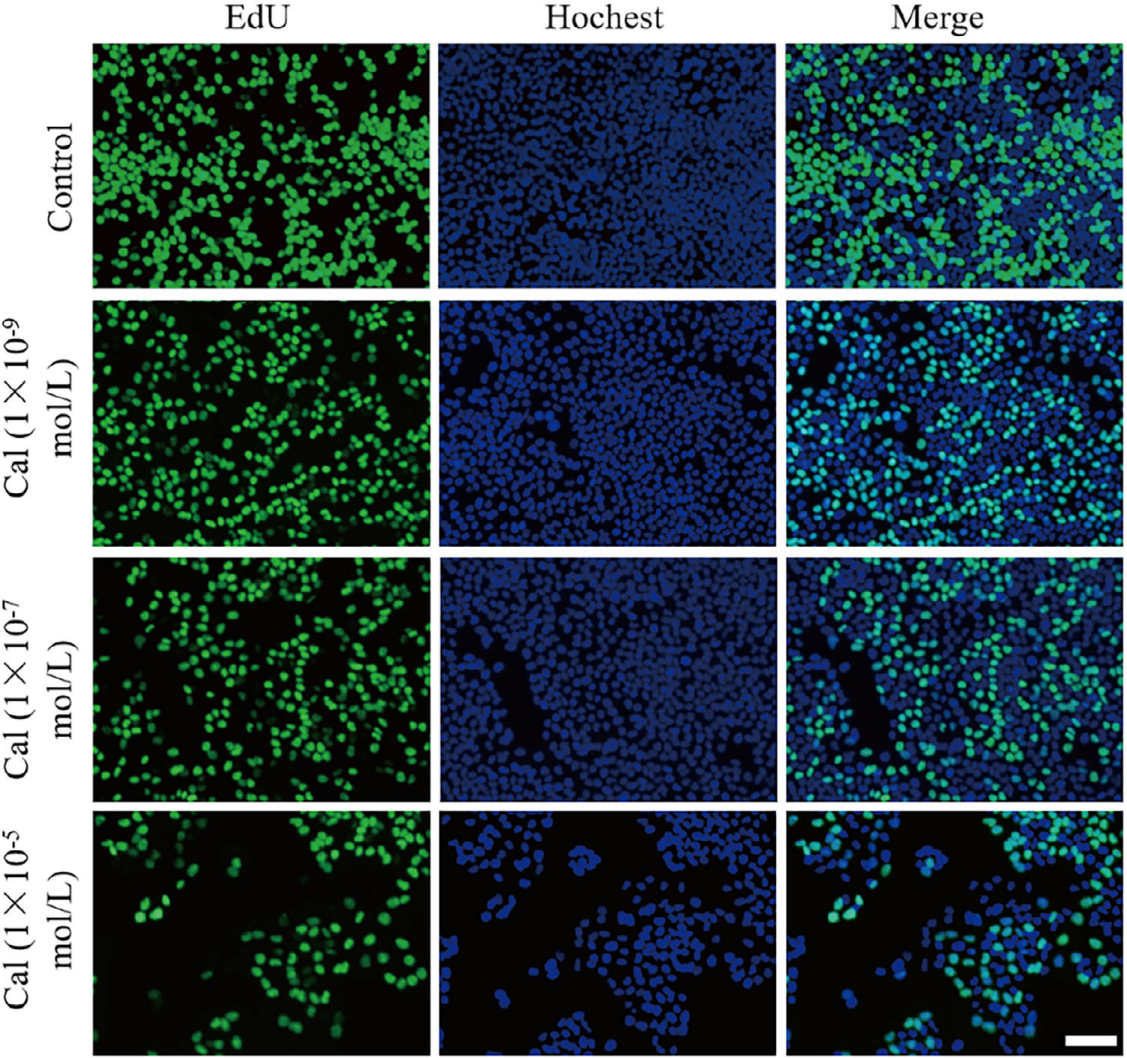


Figure S8. Relative viability of HaCaT cells incubated with extracts of various concentrations of Cal MNs after 24 h (scale bar: 100 µm).

These errors do not affect the results or conclusions of the paper.

We apologize for this error.

