# Supplementary material for: Dual‐Action Psoriasis Therapy: Antiproliferative and Immunomodulatory Effects via Self‐Locking Microneedles
Source: Adv Sci (Weinh). 2024 Oct 30;11(48):2409359. doi: 10.1002/advs.202409359 (PMC11672289; doi:10.1002/advs.202409359)
Supplement: Supplementary file 1 — Supporting Information [file ADVS-11-2409359-s001.doc]

Supporting Information

**Title: Dual-Action Psoriasis Therapy: Antiproliferative and Immunomodulatory Effects via Self-Locking Microneedles**

*Zi Yi Wang^1,2#^, Ze Qiang Zhao^3,4#^, Yu Jun Sheng^2*^, Ke Jun Chen^1,2^, Bo Zhi Chen^3,4*^, Xin Dong Guo^3,4*^, Yong Cui^1,2*^*

^1^China-Japan Friendship Hospital (Institute of Clinical Medical Sciences), Chinese Academy of Medical Sciences & Peking Union Medical College, 100029, China;

^2^Department of Dermatology, China-Japan Friendship Hospital, Beijing 100029, China;

^3^State Key Laboratory of Organic-Inorganic Composites, Beijing University of Chemical Technology, Beijing 100029, China.

^4^Beijing Laboratory of Biomedical Materials, College of Materials Science and Engineering, Beijing University of Chemical Technology, Beijing 100029, China.

Corresponding author:

cuiyong@zryhyy.com.cn (Cui, Y)

xdguo@buct.edu.cn (Guo, XD),

shengyujun@zryhyy.com.cn (Sheng, YJ),

chenbz@buct.edu.cn (Chen, BZ),

Both authors contributed equally to this work.

**A: Supporting Texts**

**Biocompatibility Evaluation of Deu@Cal MNs**

The cytotoxicity of microneedles on L-929 fibroblasts was evaluated using a CCK-8 assay. L-929 fibroblasts were initially seeded at a density of 1×10^4^ cells per well in a 96-well plate, with each well containing 100 μL of cell culture medium comprising 1% penicillin-streptomycin, 10% fetal bovine serum, and 89% DMEM. The plate was then incubated under controlled conditions (37℃, 5% CO2) for 24 hours. In co-incubation period, MNs with drug extracts of varying concentrations were introduced to each well. Following another 24-hour incubation, 10 μL of CCK-8 solution was added to each well, and the plate was incubated for an additional 3 hours. Absorbance was then measured at 450 nm using a UV-visible spectrophotometer.

The hemocompatibility of Deu@Cal MNs was assessed by examining their interaction with red blood cells (RBCs), focusing particularly on hemolysis. Blood samples were centrifuged to separate and remove plasma, and RBCs were subsequently washed three times with PBS. 5% RBC solution with MNs was then incubated at 37℃ for 1 hour. The absorbance of the supernatant at 540 nm was measured using a UV-visible spectrophotometer to quantify hemolysis. Additionally, fluorescence microscopy was used to observe RBC morphology, enabling evaluation of any structural changes.

**In Vivo Biocompatibility Evaluation of Deu@Cal MNs**

For in vivo biocompatibility assessment, healthy mice were subjected to daily treatments over 8 consecutive days with the following groups: PVA/HAMA MNs, PVA@Deu MNs, HAMA@Cal MNs, PVA@Deu/HAMA@Cal MNs. Post-treatment, mice were euthanized, and their hearts, livers, spleens, lungs, and kidneys were collected. Histological examination using hematoxylin and eosin (H&E) staining was conducted to assess potential injury in the tissues, thereby evaluating the biocompatibility of the MNs.

**Anti-proliferation Effects of Cal MNs on HaCaT cell behavior**

To examine the anti-proliferative impact of Cal MNs on keratinocytes behavior, Cal MNs were dissolved in culture medium and sterilized through filtration. This prepared medium was then co-cultured with Hacat cells. Cell proliferation and viability were assessed using EdU fluorescence staining. This method allowed for the evaluation of the anti-proliferative effects of Cal MNs on keratinocytes.

**B: Supporting Figures**


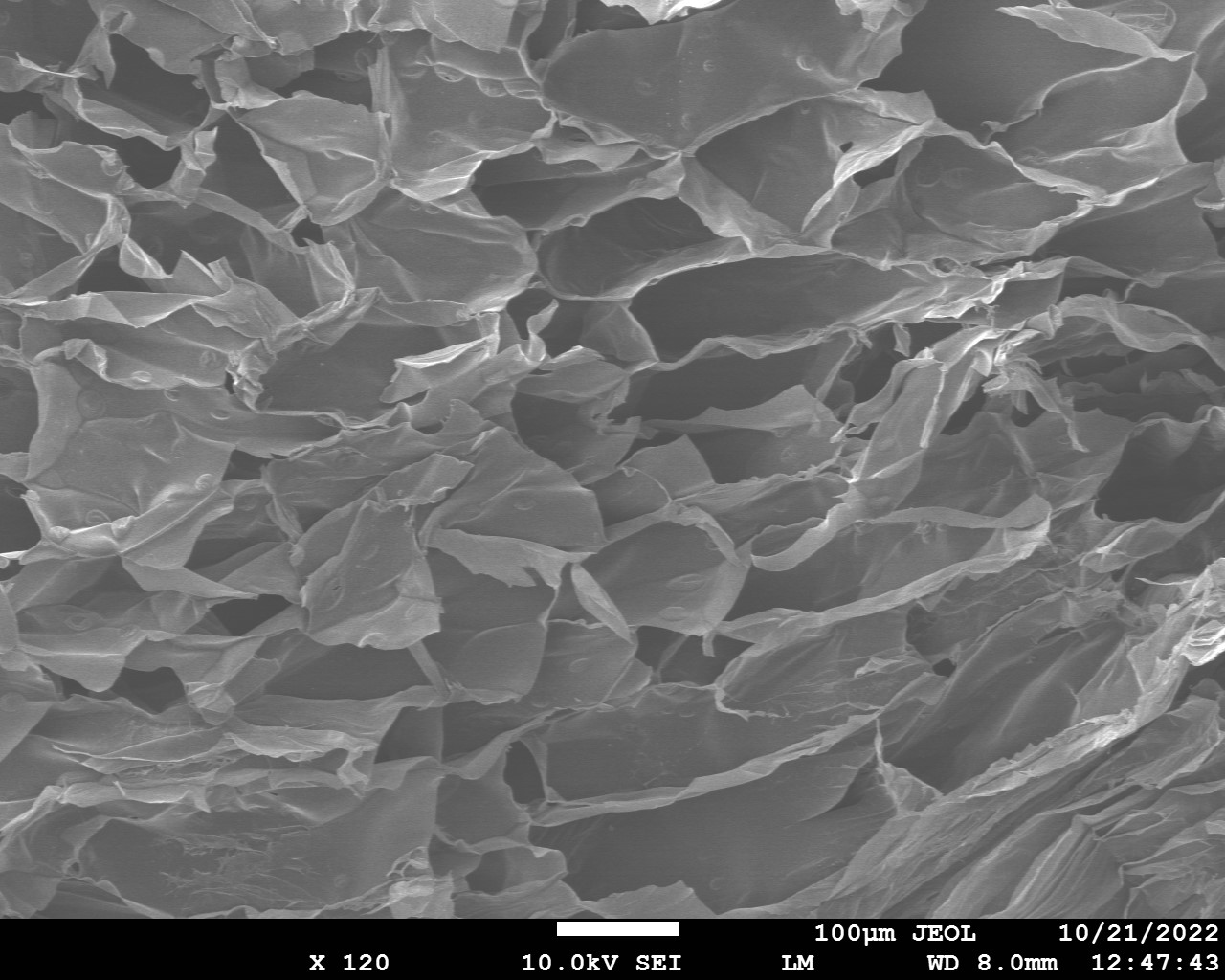

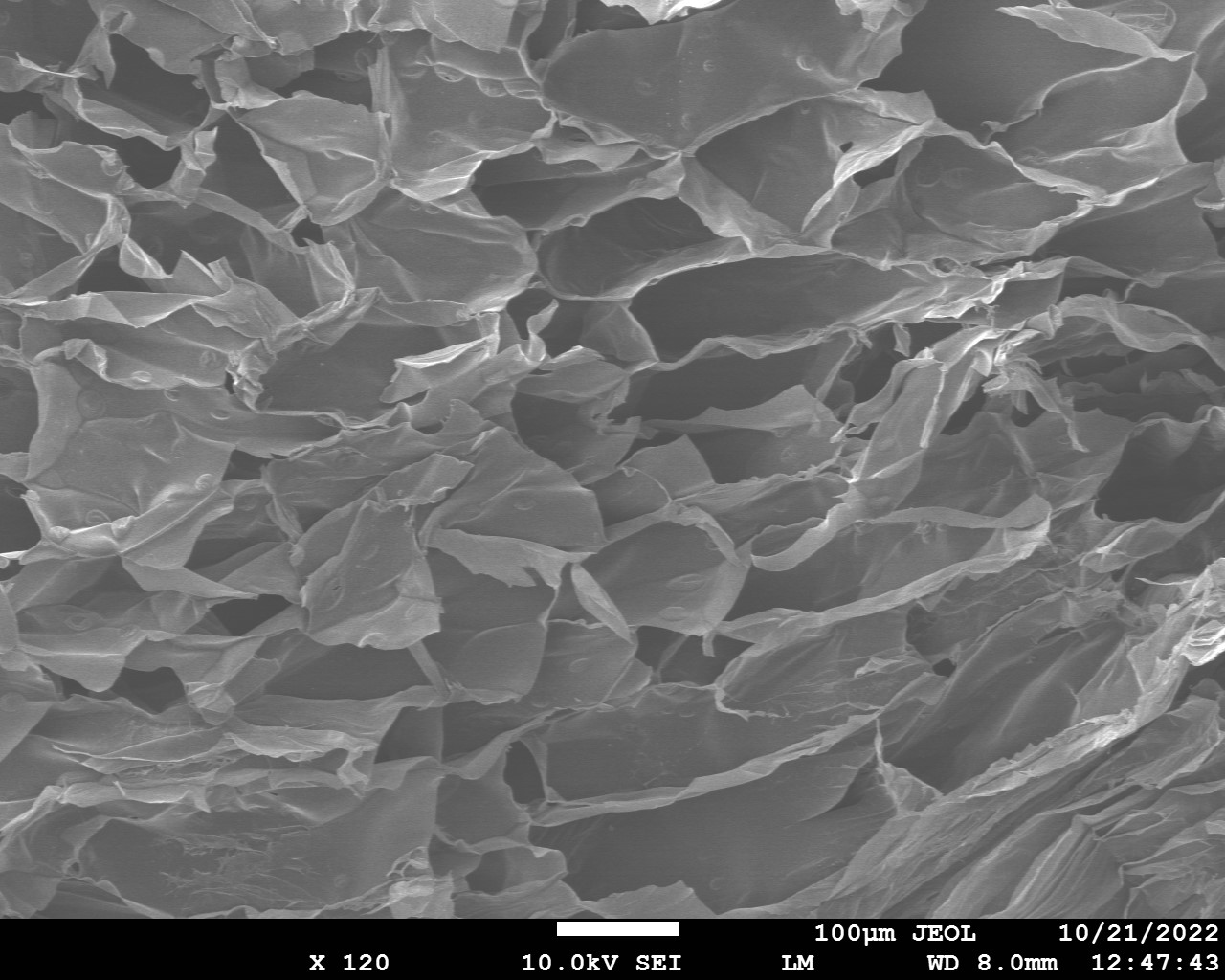


**Figure S1.** Microporous structure of HAMA materials after freeze-drying.

**
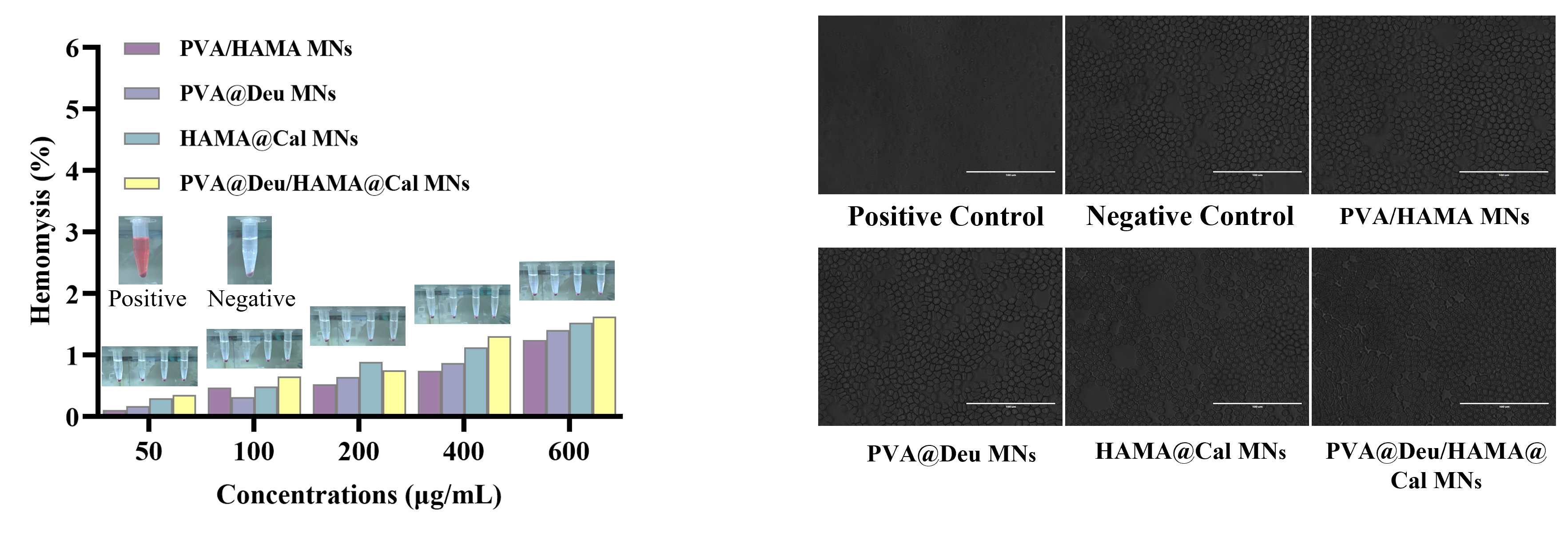
**

**Figure S2.** Blood compatibility of Deu@Cal MNs. A) Hemolysis assay results and B) corresponding microscopic images of red blood cells (scale bar: 100 μm, *n* = 5).


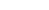

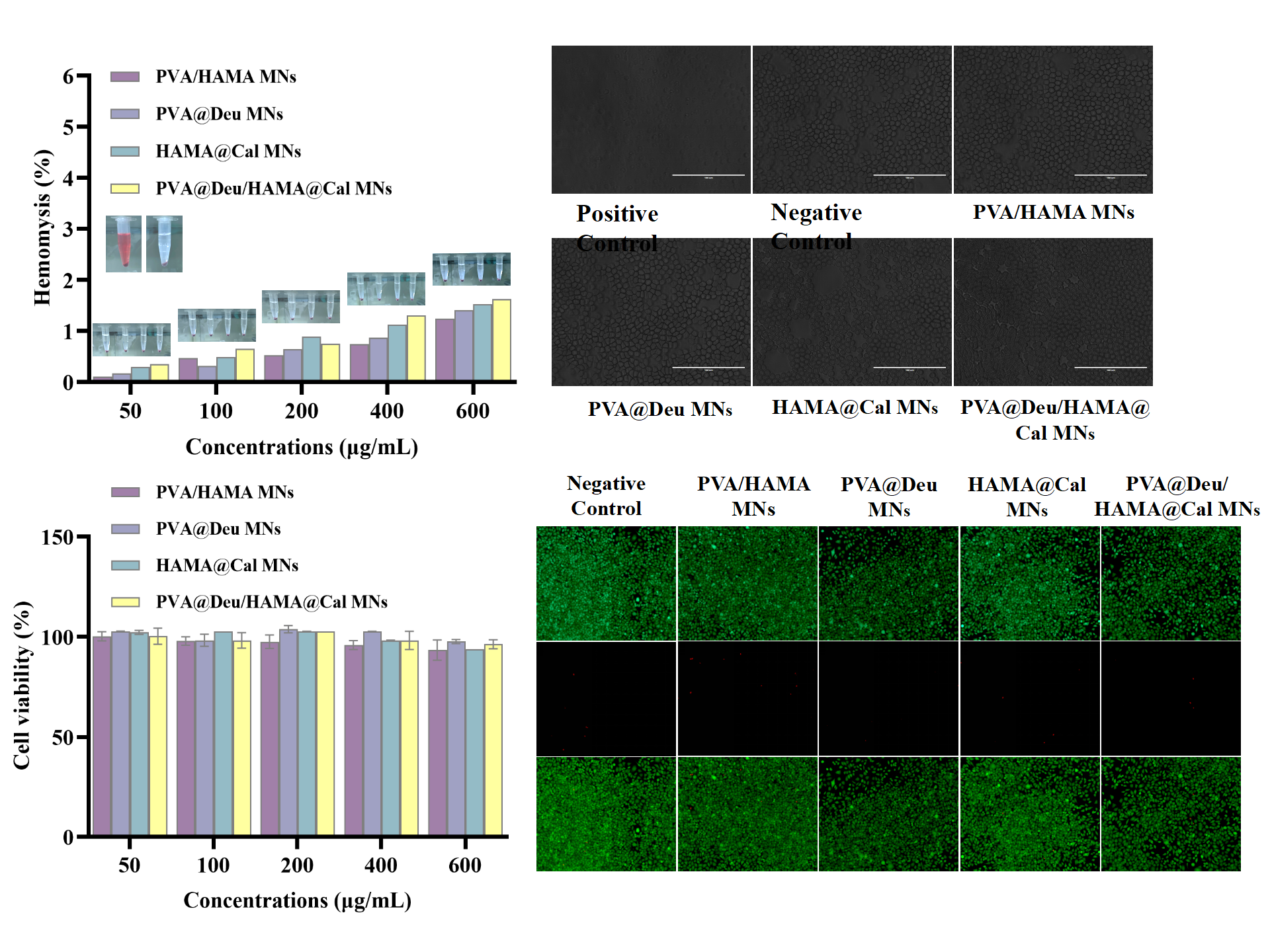


**Figure S3.** A) Relative viability of L-929 fibroblasts incubated with extracts of various concentrations of MNs after 24 h. B) Representative image of apoptosis in L-929 fibroblasts after treatment, green fluorescence for living cells, and red fluorescence for dead cells (scale bar: 100 μm).


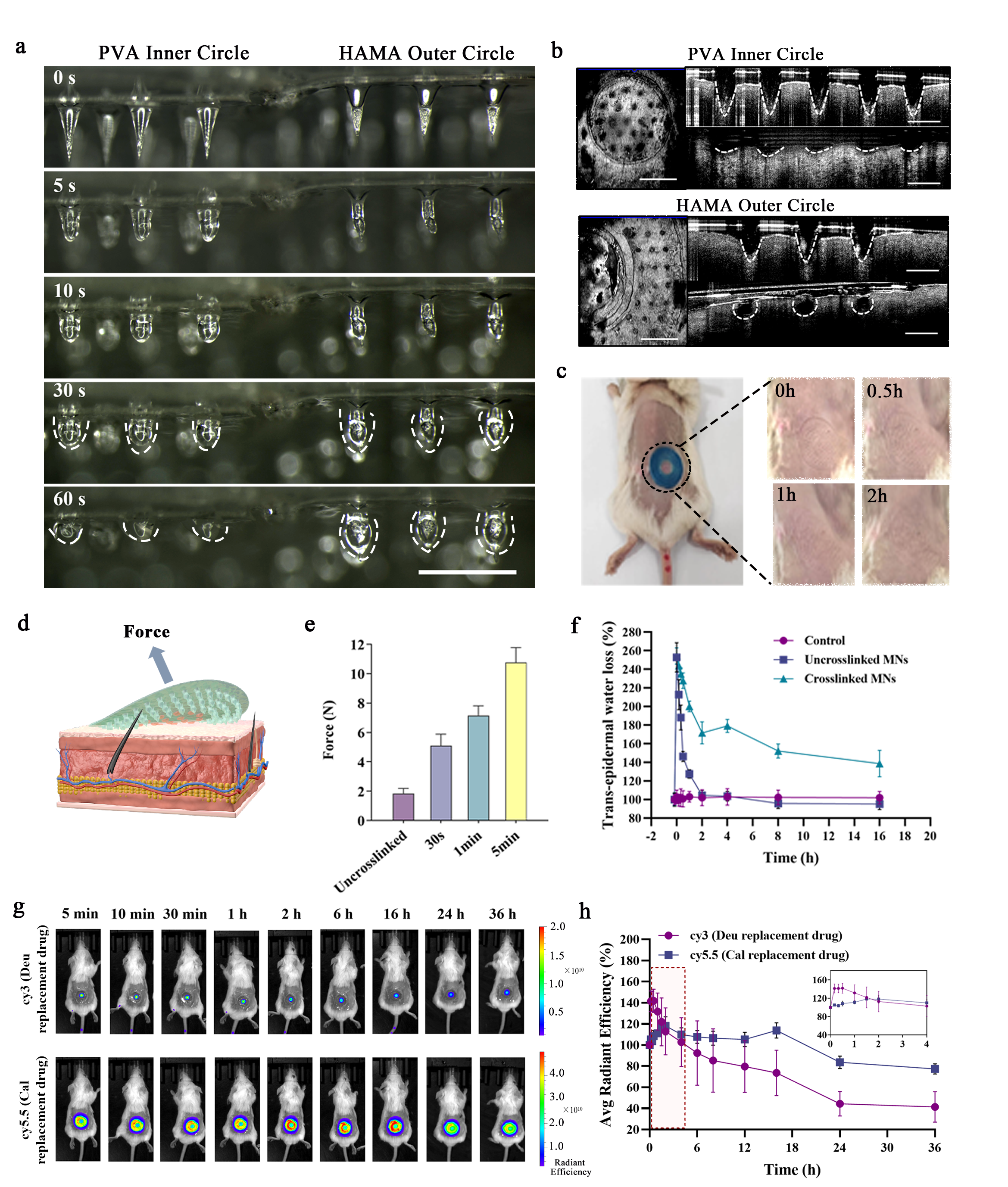


**
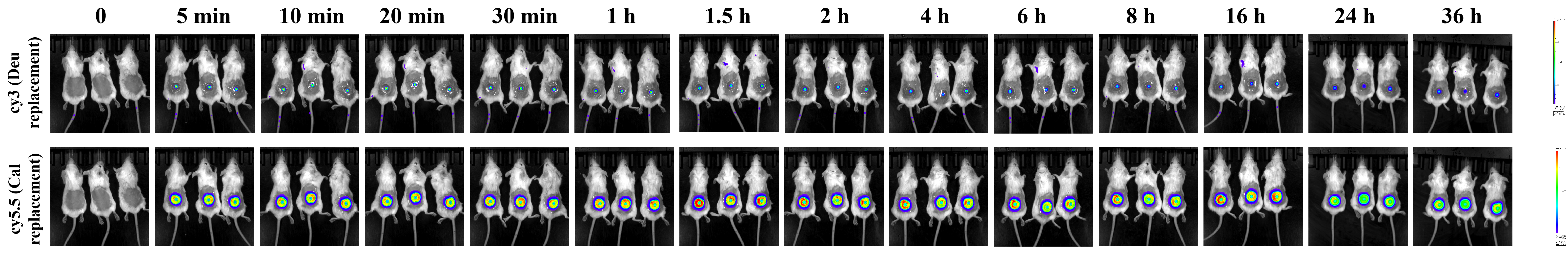
**

**Figure S4.** In vivo fluorescence images of mice at different time points after administration of the respective simulated fluorescent drugs. (cy3 as Deu replacement drug and cy5.5 as Cal replacement drug)


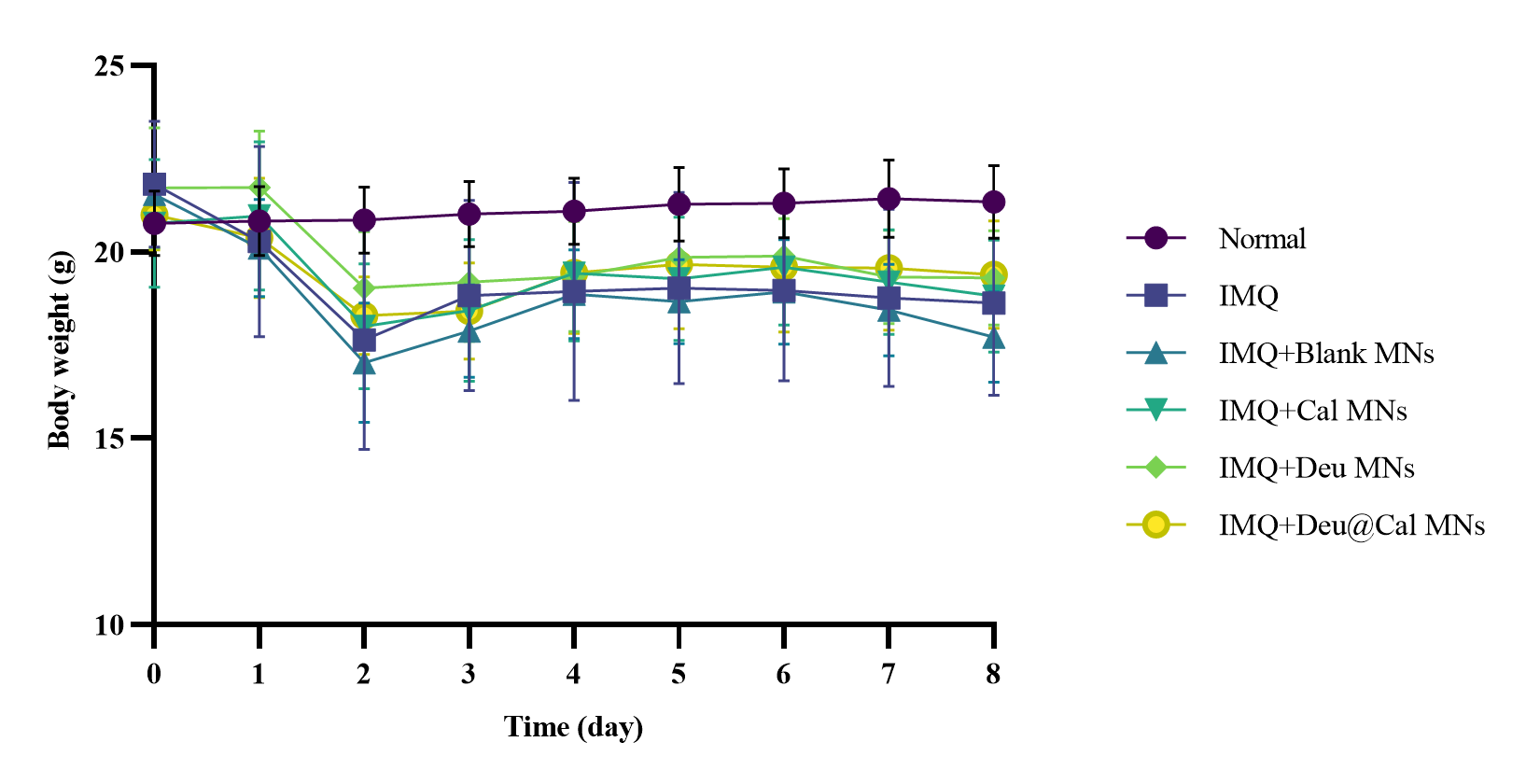


**Figure S5.** Mice body weight changes in different treatment groups. (*n*=6).

**
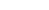

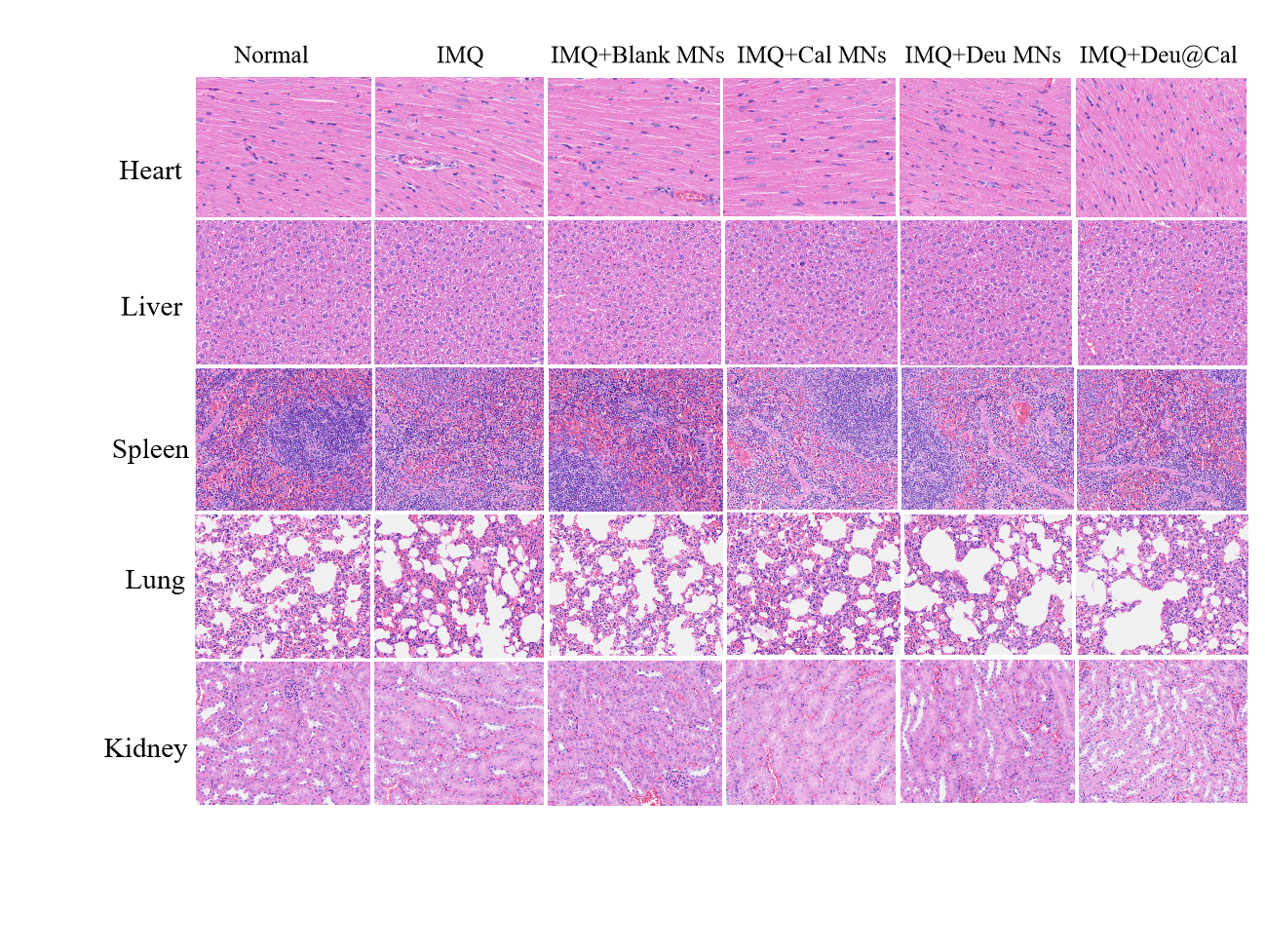
**

**Figure S6.** Representative H&E staining images of major organs in different groups (scale bar: 100 μm).

**
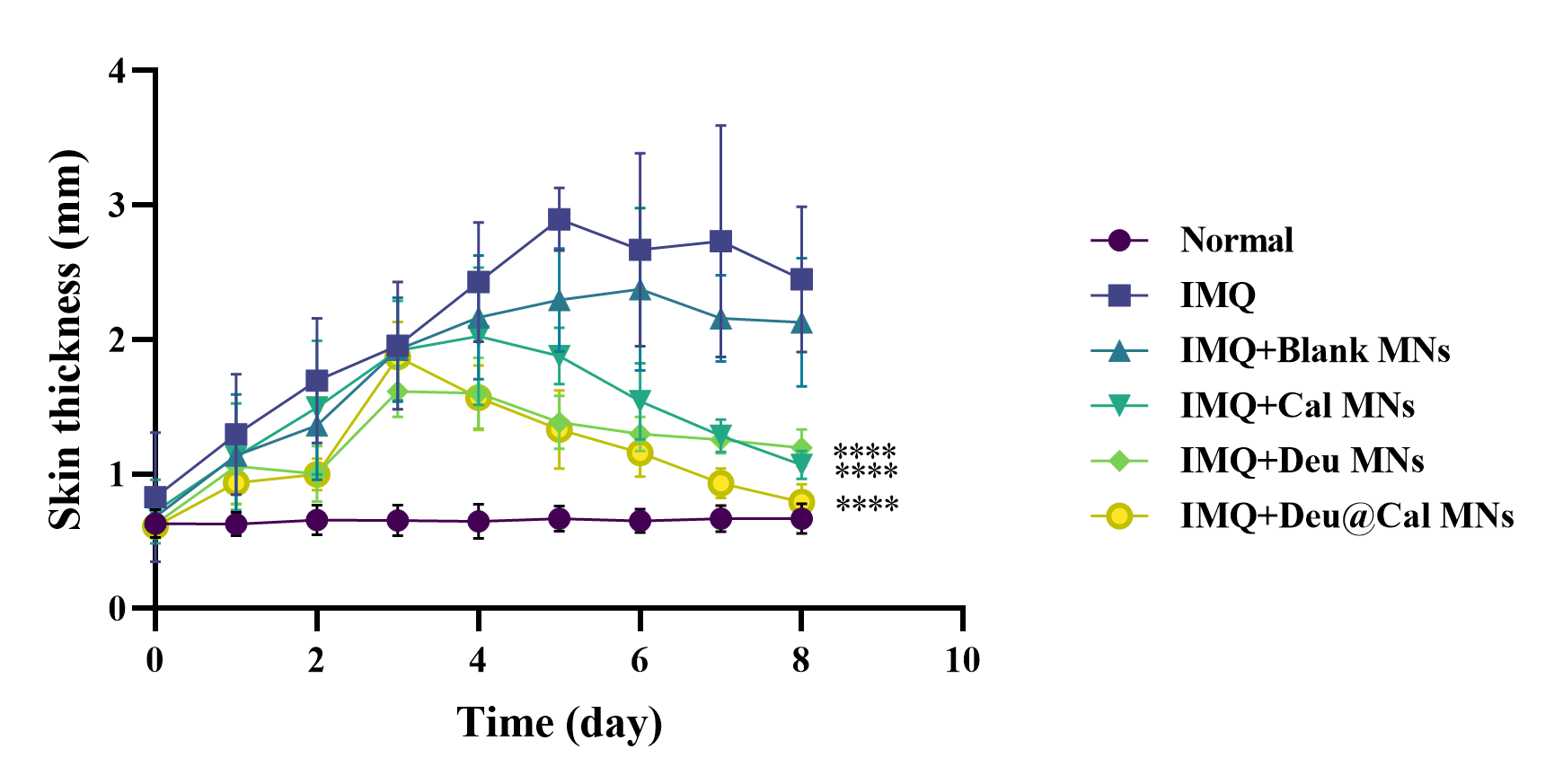
**

**Figure S7.** Measurement of dorsal skin thickness in different treatment groups. (*n*=6).


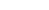
**
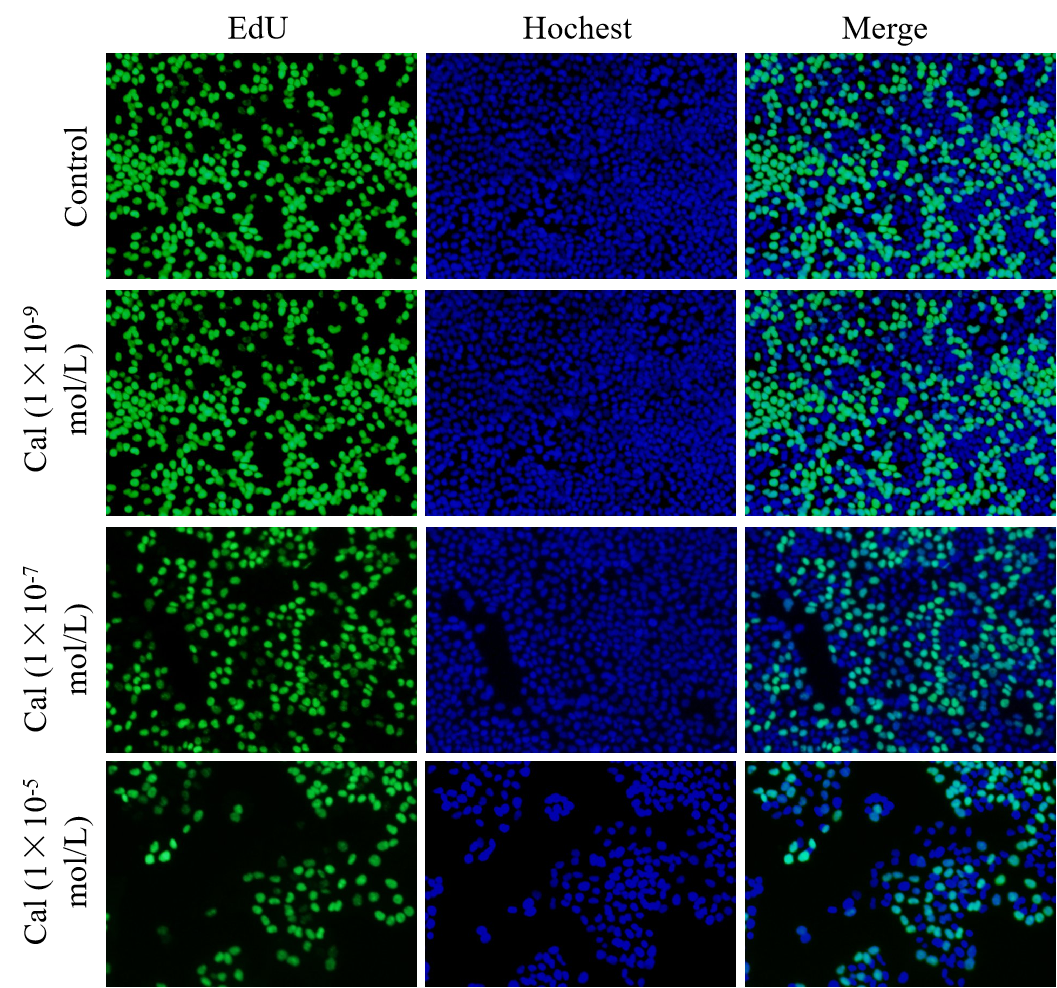
**

**Figure S8.** Relative viability of HaCaT cells incubated with extracts of various concentrations of Cal MNs after 24 h (scale bar: 100 μm).

**
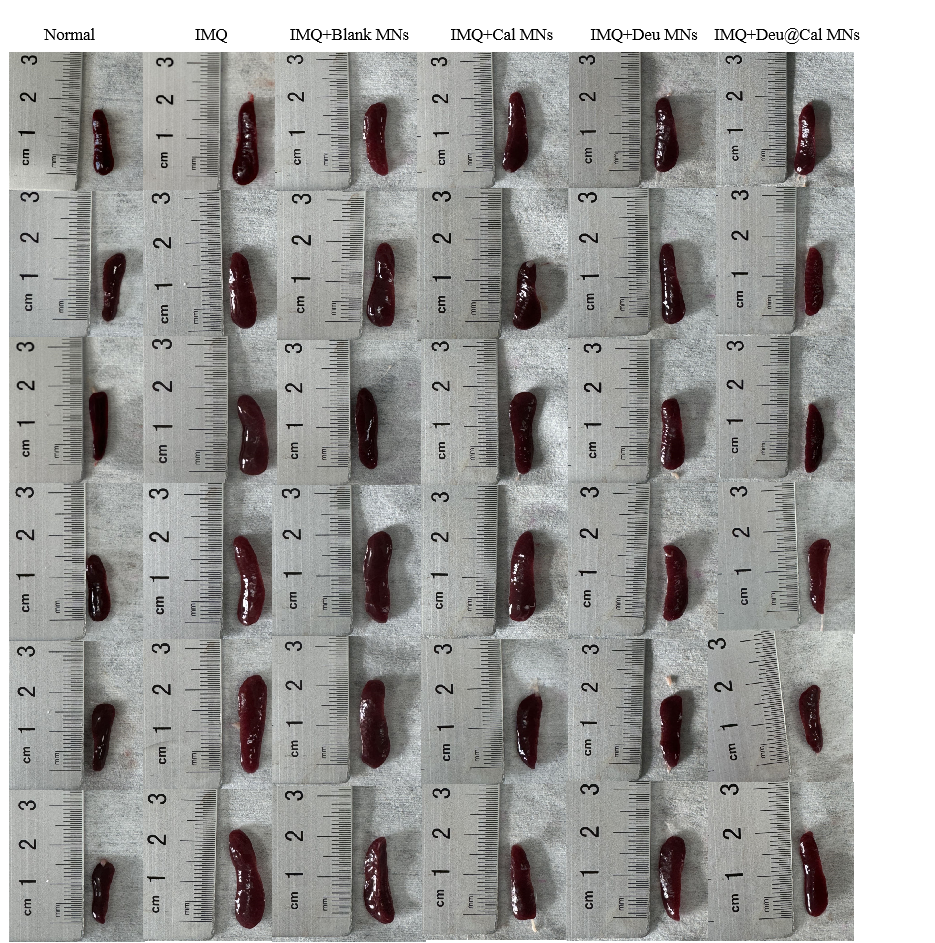
**

**Figure S9.** Mice spleen photographs after administration with different groups. (*n*=6).

**C: Supporting Tables**

**Table S1.** GO enrichment functional annotation of the up-regulated DEGs in Deu@Cal MN group.

| ONTOLOGY | ID | Description | GeneRatio | BgRatio | *p*.adjust | Count |
| --- | --- | --- | --- | --- | --- | --- |
| BP | GO:0032103 | positive regulation of response to external stimulus | 105/2661 | 458/28814 | 0.00000000 | 105 |
| BP | GO:0002697 | regulation of immune effector process | 101/2661 | 436/28814 | 0.00000000 | 101 |
| BP | GO:0050900 | leukocyte migration | 95/2661 | 380/28814 | 0.00000000 | 95 |
| MF | GO:0005543 | phospholipid binding | 84/2602 | 459/28275 | 0.00000022 | 84 |
| BP | GO:0060326 | cell chemotaxis | 83/2661 | 314/28814 | 0.00000000 | 83 |
| BP | GO:0002699 | positive regulation of immune effector process | 78/2661 | 309/28814 | 0.00000000 | 78 |
| BP | GO:0030595 | leukocyte chemotaxis | 65/2661 | 228/28814 | 0.00000000 | 65 |
| CC | GO:0005774 | vacuolar membrane | 61/2674 | 239/28739 | 0.00000000 | 61 |
| MF | GO:0030246 | carbohydrate binding | 55/2602 | 277/28275 | 0.00000870 | 55 |
| CC | GO:0005765 | lysosomal membrane | 50/2674 | 187/28739 | 0.00000000 | 50 |
| CC | GO:0098852 | lytic vacuole membrane | 50/2674 | 187/28739 | 0.00000000 | 50 |
| CC | GO:0044391 | ribosomal subunit | 48/2674 | 195/28739 | 0.00000003 | 48 |
| CC | GO:0022626 | cytosolic ribosome | 45/2674 | 111/28739 | 0.00000000 | 45 |
| MF | GO:0003735 | structural constituent of ribosome | 44/2602 | 164/28275 | 0.00000002 | 44 |
| MF | GO:0140375 | immune receptor activity | 43/2602 | 136/28275 | 0.00000000 | 43 |
| MF | GO:0019955 | cytokine binding | 35/2602 | 143/28275 | 0.00001120 | 35 |
| MF | GO:0004896 | cytokine receptor activity | 32/2602 | 97/28275 | 0.00000002 | 32 |
| CC | GO:0022627 | cytosolic small ribosomal subunit | 21/2674 | 46/28739 | 0.00000002 | 21 |

**Table S2.** Primers for qPCR

| Gene | Forward | Reverse |
| --- | --- | --- |
| GADPH | CATCACTGCCACCCAGAAGACTG | ATGCCAGTGAGCTTCCCGTTCAG |
| IL-6 | CCTCTCTGCAAGAGACTTCCAT | AGTCTCCTCTCCGGACTTGT |
| IL-17 | CAGACTACCTCAACCGTTCCAC | TCCAGCTTTCCCTCCGCATTGA |
| IL-23 | TCCTCCAGCCAGAGGATCACCC | AGAGTTGCTGCTCCGTGGGC |
| TNF-α | ACGCTCTTCTGTCTACTGAACTTC | GGTTTGTGAGTGTGAGGGTCTG |
